# Induced Pluripotent Stem Cell‐Derived Parathyroid Organoids Resemble Parathyroid Morphology and Function

**DOI:** 10.1002/advs.202407567

**Published:** 2024-09-27

**Authors:** Selinay Şenkal‐Turhan, Ezgi Bulut‐Okumuş, Muhterem Aydın, Neşe Başak Türkmen, Aslı Taşlıdere, Fikrettin Şahin, Şahin Yılmaz, Pınar Akkuş Süt, Ayşegül Doğan

**Affiliations:** ^1^ Department of Genetics and Bioengineering Faculty of Engineering Yeditepe University İstanbul 34755 Turkey; ^2^ Department of Veterinary Obstetrics and Gynecology Faculty of Veterinary Medicine University of Fırat Elazığ 23119 Turkey; ^3^ Department of Pharmaceutical Toxicology Faculty of Pharmacy University of Inonu Malatya 44280 Turkey; ^4^ Department of Histology and Embryology Faculty of Medicine University of Inonu Malatya 44280 Turkey

**Keywords:** 3D culture, hypoparathyroidism, organoid, parathyroid, parathyroid organoid, pluripotent stem cell

## Abstract

The primary role of the parathyroid glands is to maintain calcium homeostasis through the secretion of parathyroid hormone (PTH). The limited proliferative capacity and differentiation of parathyroid cells hinder the generation of cell therapy options. In this study, parathyroid organoids are successfully generated from human‐induced pluripotent stem cells (hiPSCs). At the end of the 20 days of differentiation, the parathyroid organoids exhibited distinct parathyroid morphology. Stereomicroscope, scanning electron microscopy (SEM), and transmission electron microscopy (TEM) analysis demonstrated the 3D arrangement of the cell layers in which intracellular structures of parathyroid cells resemble human parathyroid cellular morphology. Comprehensive molecular analyses, including RNA sequencing (RNA‐Seq) and liquid chromatography/mass spectrometry (LC‐MS/MS), confirmed the expression of key parathyroid‐related markers. Protein expression of CasR, CxCr4, Gcm2, and PTH are observed in parathyroid organoids. Parathyroid organoids secrete PTH, demonstrate active intercellular calcium signaling, and induce osteogenic differentiation via their secretome. The tissue integration potential of parathyroid organoids is determined by transplantation into parathyroidectomized rats. The organoid transplanted animals showed significant elevations in PTH‐related markers (CasR, CxCr4, Foxn1, Gcm2, and PTH). PTH secretion is detected in organoid‐transplanted animals. The findings represent a significant advancement in parathyroid organoid culture and may offer a cellular therapy for treating PTH‐related diseases, including hypoparathyroidism.

## Graphical abstract

iPSC‐derived parathyroid organoids express PTH‐related markers at the protein and gene levels. The transplantation of parathyroid organoids into parathyroidectomized rats shows their functional activity and tissue integration capability. iPSC‐derived parathyroid organoids successfully release PTH both in vitro and in vivo. Parathyroid organoid technology may improve defective parathyroid function and related diseases.

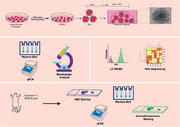

## 1 Introduction

The parathyroid glands are endocrine organs that play a key role in regulating calcium levels in the body by releasing parathyroid hormone (PTH), which acts on the bone, kidney, and small intestine.^[^ ^1^, ^2^ ^]^ The glands consist of chief cells, which control the level of calcium ions in the bloodstream by secreting PTH, and oxyphil cells, whose function is not clearly determined.^[^ ^3^ ^]^ Defects in parathyroid gland function, such as hyperparathyroidism or hypoparathyroidism, disrupt the calcium homeostasis and may potentially necessitate surgical treatment of parathyroid glands. Developing parathyroid culture models that accurately replicate the physiology and function of the organ is crucial for understanding the molecular mechanisms behind parathyroid homeostasis.^[^ ^4^ ^]^

Advocating for significant improvements in stem cell research, scientists have created 3D organ‐like structures known as organoids.^[^ ^5^ ^]^ Organoids can be formed using embryonic stem cells (ESCs), induced pluripotent stem cells (iPSCs), or isolated organ progenitors from neonatal, adult, or fetal sources.^[^ ^6^ ^]^ The purpose of these organoids is to study the molecular, structural, and functional characteristics of organs and tissues in a controlled environment by replicating the conditions found in living organisms.^[^ ^7^, ^8^ ^]^ As J. Kim et al.^[^ ^9^ ^]^ review, numerous well‐established organoid models exist for various organs including different types of endocrine and exocrine glandular tissues.^[^ ^10^, ^11^, ^12^ ^]^ Until now, the organoid models have provided remarkable insights into human development and organ regeneration, highlighting their significance for biology research, drug testing, and molecular medicine.^[^ ^9^ ^]^ It is possible to create an organoid that resembles the structure of the mature organ by mimicking the developmental process in pluripotent stem cell (PSC) culture.

Compared to the generation of complex organ‐like structures, formation of organoid models for small endocrine organs, like the parathyroid glands, and transplantation of these organoids, can be considered more straightforward. Derivation of parathyroid organoids using pluripotent stem cells, including the ones of patients’ own iPSCs, might be a promising option for further clinical applications, organ development, disease modeling, and drug screening. Prior studies have focused on acquiring parathyroid cells from ESCs.^[^ ^13^ ^]^ However, the most recent research has made progress in generating parathyroid organoids using parathyroid‐like cells produced from human ESCs.^[^ ^14^ ^]^ Here, parathyroid organoids were generated using fibroblast‐derived iPSCs and characterized both in vitro and in vivo. There is evidence of gene and protein expression, hormone release, and in vitro functional activity. Molecular analyses have revealed the gene pathways that control parathyroid differentiation. The in vivo functional activity of organoids was proven using a rat model of parathyroidectomy. In this manner, the resulting 3D organoid structures were characterized in vitro and in vivo, which are actual physiological environmental conditions.

## 2 Results

### 2.1 Derivation and Characterization of iPSCs

**Figure 1:**
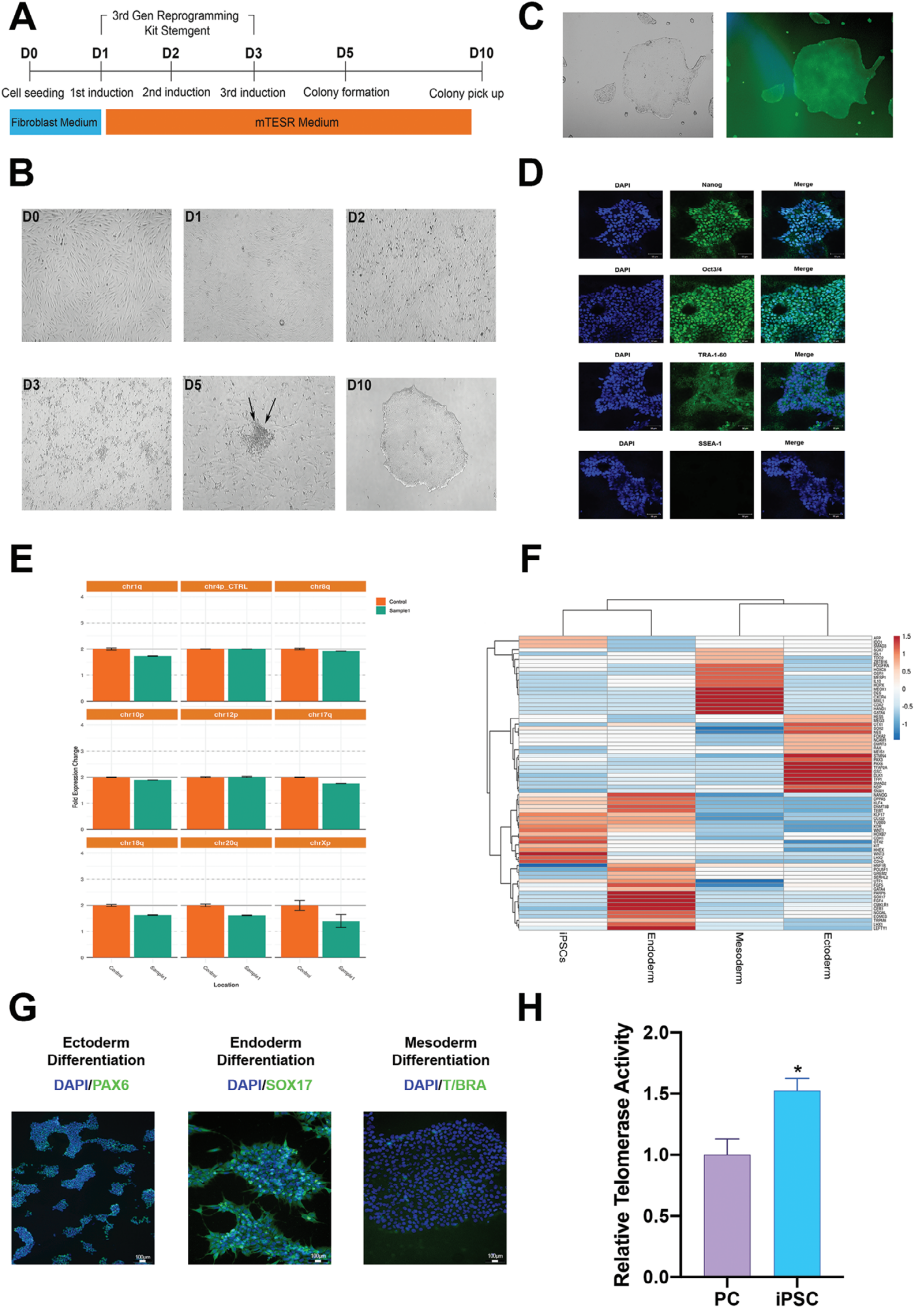
The generation and characterization of iPSCs from human dermal fibroblasts. A) The experimental methodology employed for the generation of iPSCs by reprogramming of human fibroblasts using the Stemgent StemRNA 3rd Gen Reprogramming Kit. B) After the process of induction, fibroblast cells exhibited morphological alterations, and the formation of colonies became apparent within 5 days. C) The ALP activity staining was performed on representative colonies of iPSCs using a fluorescence microscope. D) The characterization of iPSCs involved detection of the pluripotency markers, including Nanog, Oct3/4, TRA1‐60, and SSEA‐1. Oct3/4: Octamer‐binding transcription factor ¾, TRA1‐60: Tumor resistance antigen 1–60, SSEA‐1: Stage‐specific embryonic antigen‐1. n = 3, Scale bar: 50 µm. E) The evaluation of chromosomal aberrations during the reprogramming of iPSCs was conducted using qPCR analysis. F) The hierarchical clustering of iPSC (pluripotency) and three germ layers (ectoderm, endoderm, and mesoderm) related genes. G) Immunostaining of PAX6 (ectoderm), Sox17 (endoderm), and T/Bra (mesoderm) markers was conducted using fluorescence microscopy on cells that had undergone differentiation. n = 3. H) Telomerase activity of iPSCs compared to PC detecting by telomerase activity quantification qPCR assay kit. Data presented as mean ± SEM, n = 3, and p‐values are calculated using an unpaired t‐test, *p < 0.05.

HDFCs were successfully reprogrammed into iPSCs using the StemRNA 3rd Generation Reprogramming Kit (**Figure** **1A**). iPSC colonies began to appear at day five. Alkaline phosphatase (ALP)‐positive colonies were selected on day 10 for further experiments (Figure 1C). iPSCs were positive for pluripotency markers such as Nanog, Oct3/4, and TRA1‐60, and negative for SSEA‐1 (Figure 1D). There was no chromosomal abnormality for the indicated chromosomal areas (Figure 1E). The 3‐germ layer differentiation capacity of iPSCs were demonstrated by gene expression and immunostaining analysis. Gene expression profile revealed a distinct separation between control cells and differentiated cells (endoderm, ectoderm, and mesoderm). Markers such as PAX6 (ectoderm), Sox17 (endoderm), and T/Bra (mesoderm) were positive in the differentiated cells (Figure 1G; Figure S1, Supporting Information). Distinct cellular morphologies of 3‐germ layers were observed in each group (Figure S1B, Supporting Information). Telomerase activity of the iPS cells was 1.6 times more than that of the positive control (Figure 1H).

### 2.2 Parathyroid Organoid Generation and Characterization

**Figure 2:**
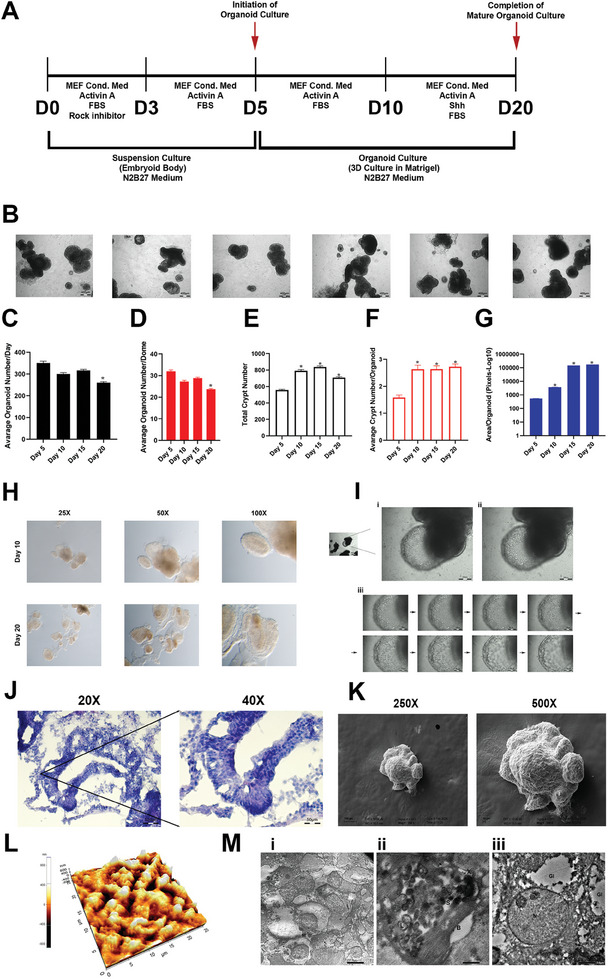
Generation of parathyroid organoids derived from iPSCs. A) The schematic representation of parathyroid organoid generation protocol. B) An illustrative morphological image of parathyroid organoids acquired on the twentieth day. Scale bar: 400 µm, n = 3. C) Daily average number of organoids. *p < 0.05, n = 3. D) Mean number of organoids per dome. *p < 0.05, n = 3. E) Total quantity of crypts. *p < 0.05, n = 3. F) Mean crypt counts per organoid. *p < 0.05, n = 3. G) Area of organoids (Pixels‐Log10). *p < 0.05, n = 3. H) Stereomicroscope images of representative parathyroid organoids and crypt structures obtained on days 10 and 20, respectively. I) Light microscope analysis of organoids (i) the outer and (ii) inner cell layering of crypts that branch off (iii) morphological examination of crypt cell layers, focused from the center to the wall. Scale bar: 400 µm. J) Histological examination of parathyroid organoid slices at different magnifications with a thickness of 10 µm, stained with H&E. Scale bar: 100 µm, 50 µm, n = 3. K) Scanning electron microscopy image of parathyroid organoids at various magnifications. Scale bar: 100 µm, 10 µm. L) AFM analysis of parathyroid organoids. M) Transmission electron microscopy image of parathyroid organoids. (i) Dispersed secretion granules (S) and mitochondria (M) of varying sizes, with numerous small vesicles and granules of varying densities (illustrated by arrows). (ii) The cytoplasm of the cells contains significant amounts of lipid (L), and scattered secretion granules (S). The collection of cells is surrounded by a unique basement membrane (B). The internal portion of the granule consists of compact, rod‐shaped structures (X), which, in certain regions, arrange themselves in a manner that resembles the packing of crystals. (iii) The cytoplasm of the cells contains significant amounts of glycogen (Gl) and cells including the nucleus (N).

Parathyroid organoids were generated using a 20‐day differentiation protocol and a specific culture medium (Table S1, Supporting Information). The schematic representation of the 20‐day protocol for parathyroid organoid generation is shown in **Figure** **2**A. The 3D morphological structure and crypt‐like characteristics of parathyroid organoids were clearly observed at day 20 (Figure 2B). Over the duration of a 20‐day differentiation period, there was a notable rise in the numbers of organoids seen on a daily basis, as well as an increase in the number of organoids within the same dome (Figure 2C, D). During the process of differentiation, there was a significant increase in both the overall crypts number in organoid culture and the number of crypts per organoid (Figure 2E,F). Furthermore, there was an important increase in the area of the organoids over the course of the 20‐day differentiation period, suggesting that the culture of organoid may progress without the disruption 3D structure (Figure 2G).

The macroscopic visualization of the internal structure of the parathyroid organoids using stereomicroscope enabled the observation of discernible morphologies reminiscent of parathyroid tissue at day 10 and 20 (Figure 2H). Cellular organization of the crypt structure showed a 3D cell layer arrangement (Figure 2I). The Hematoxylin‐Eosin (H&E) staining of the organoids exhibited a shape resembling that of typical parathyroid tissue (Figure 2J). Furthermore, scanning electron microscopy (SEM) (Figure 2K; Figure S2, Supporting Information) allowed for the observation of the 3D structure of parathyroid organoids and the branching on their surface. The atomic force microscopy (AFM) clearly revealed the surface roughness and structural protrusions of the parathyroid organoids (Figure 2L; Figure S3, Supporting Information). In transmission electron microscopy (TEM) analysis, active chief cells are distinguished by their little cytoplasmic glycogen and many secretory granules (Figure 2K). The human parathyroid cells are enveloped by a separate basement membrane (Figure 2M), where collagen fibers are embedded. We have not detected any ergastoplasm or Golgi membranes in the oxyphil cells (Figure 2M). Figure S4 (Supporting Information) illustrates a side‐by‐side comparison of a single organoid that are embedded as a one single organoid and multiple organoids within a dome structure. The data indicates that the process of differentiating numerous organoids is more efficient compared to differentiating a single organoid (Figure S4, Supporting Information). This finding implies that there may be paracrine interactions occurring through the organoid secretome.

**Figure 3:**
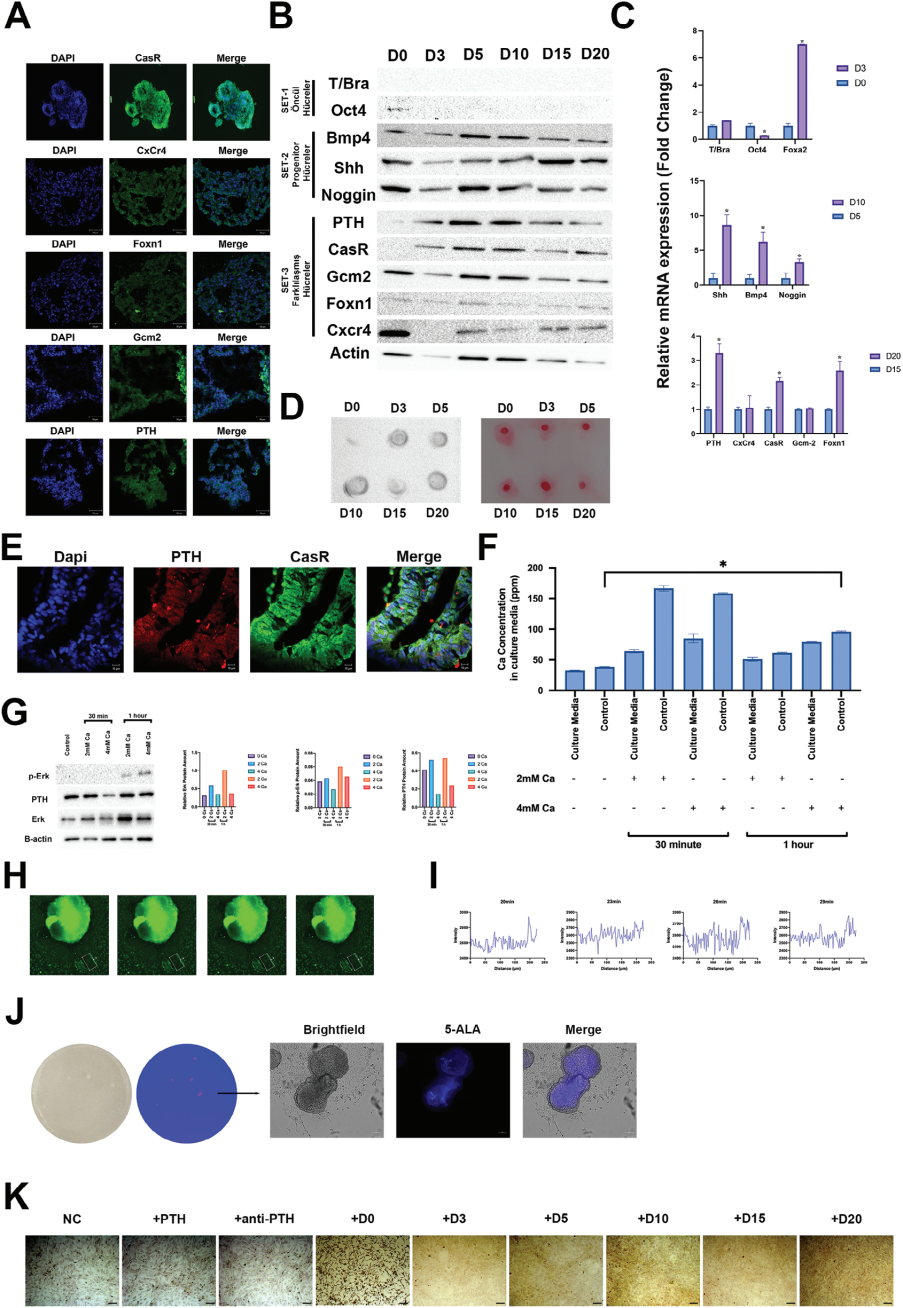
The establishment of functional parathyroid organoids in vitro. A) The immunofluorescence staining images reveal the cellular translocation of CasR, CxCr4, Foxn1, Gcm2, and PTH in parathyroid organoids at day 20. Scale bar: 50 µm, n = 3. B) Western blot analysis of organoids. T/Bra, Oct4, Bmp4, Shh, Noggin, PTH, CasR, Gcm2, Foxn1, and Cxcr4 proteins expression analysis, n = 3. C) The relative mRNA expressions of three sets of genes were analyzed in individual sets: D0‐D3: (T/Bra, Oct4, Foxn1); D5‐D10: (Shh, Bmp4, Noggin); and D15‐D20: (PTH, CxCr4, CasR, Gcm‐2). Data presented as mean ± SEM, n = 3, and p‐values are calculated using one‐way ANOVA, *p < 0.05. D) Dot blot analysis of PTH and Ponceau S stain for total protein. E) Double staining of PTH and CasR in organoids at Day 20. Scale bar: 10 µm. F) The analysis of calcium concentrations by ICP‐MS in cell culture media in response to different calcium concentrations in a time‐dependent manner. Data presented as mean ± SEM, n = 3, and p‐values are calculated using one‐way ANOVA, *p < 0.05. G) Western blot analysis of Erk, p‐Erk, and PTH in calcium‐treated organoids. (H) The Fluo‐4 recordings of Ca^2+^ transfer in one region (indicated rectangles) of parathyroid organoids. I) The graphs of Fluo‐4 recordings of distance versus intensity every 3 minutes. J) The fluorescence signal response of organoids to 5‐ALA administration. Scale bar: 500 µm. K) Alizarin red staining of mineralized areas. Scale bar: 200 µm.

The functional evaluation of parathyroid organoids revealed positive expression of parathyroid markers, including CasR, CxCr4, Foxn1, Gcm2, and PTH at day 20 (**Figure** **3**A). Concurrently, the precursor groups in the differentiation process exhibited positive immunostaining of the relevant genes (Figures S5–S9, Supporting Information). We observed positive Oct4 protein expression in our pluripotent stem cell (Figure S5, Supporting Information). Our differentiated cells showed protein‐level expression of the Foxa2 (definitive endoderm) marker on the third day (Figure S6, Supporting Information). Shh, Noggin, and BMp4 exhibited positive expression in cell groups at days 5 and 10 as differentiation markers of the parathyroid developmental process (Figures S7 and S8, Supporting Information). On the 15th day, the cell groups positively expressed the parathyroid markers CasR, CxCr4, Gcm2, and PTH, while Foxn1, the thymus marker, showed a slight positive expression (Figure S9, Supporting Information). Specifically, there was no statistically significant rise in PTH‐related proteins seen on days 15 and 20 (Figure 3B). Upon analyzing the markers at the gene level, it was observed that the levels of Foxa2 considerably increased on day 3. Similarly, the levels of Shh, Bmp4, and Noggin showed a large increase on day 10. Additionally, the levels of CasR, Foxn1, and PTH exhibited a significant rise on day 20 (Figure 3C). The existence of the PTH protein was further verified using dot‐blot analysis. While the expression levels of PTH varied on different days, the concentration of PTH was higher than the total protein concentration on the 10th and 20th days (Figure 3D). The localization of PTH‐CasR double staining in parathyroid organoids was confirmed to be positive (Figure 3E; Figure S10, Supporting Information).

### 2.3 Functionality Validation of Parathyroid Organoids

The calcium content in the culture media of organoids was measured by the exposure of different calcium concentrations (2 mM, 4 mM) in a time‐dependent manner (30 min, 1 h). The maximum calcium concentration was determined when the parathyroid organoids were exposed to 2 mM calcium for 30 min (Figure 3F). In the groups that received calcium for one hour, an increase in PTH, Erk, and p‐Erk protein levels was observed (Figure 3G). Calcium transport in organoids was shown in Figure 3H. The transfer of the calcium signal was obvious in the distance‐intensity graphs (Figure 3I; Figure S11, Supporting Information). Furthermore, when parathyroid organoids were exposed to cinacalcet and various calcium concentrations (2, 4 mM), the levels of calcium and phosphate in the cell culture media were reduced in the groups treated with 4 mM calcium and cinacalcet, compared to the control (only 4 mM calcium), as expected (Figure S12A, Supporting Information). Furthermore, there was no notable difference in the number of viable cells detected in organoids subjected to cinacalcet and calcium, indicating that the drug did not have a negative impact on cell viability (Figure S12B, Supporting Information). 5‐Aminolevulinic acid (5‐ALA), which is used for red fluorescent detection of parathyroid tissue in response to 405 nm, also worked in parathyroid organoids in vitro (Figure 3J). The addition of conditioned media derived from organoids at days 0 and 20, and the addition of PTH hormone to the culture medium increased in vitro bone differentiation of MC3T3‐E1 preosteoblast cells (Figure 3K; Figure S13B, Supporting Information). The protein expressions of Col1 and OC were higher in the D0 conditioned media and PTH‐applied groups (Figure S13A, Supporting Information). The day 20 conditioned media group induced the expression of the osteogenic marker genes compared to control (Figure S13C, Supporting Information).

### 2.4 Characterization of iPSC‐Derived Parathyroid Organoids at the Molecular Level

**Figure 4:**
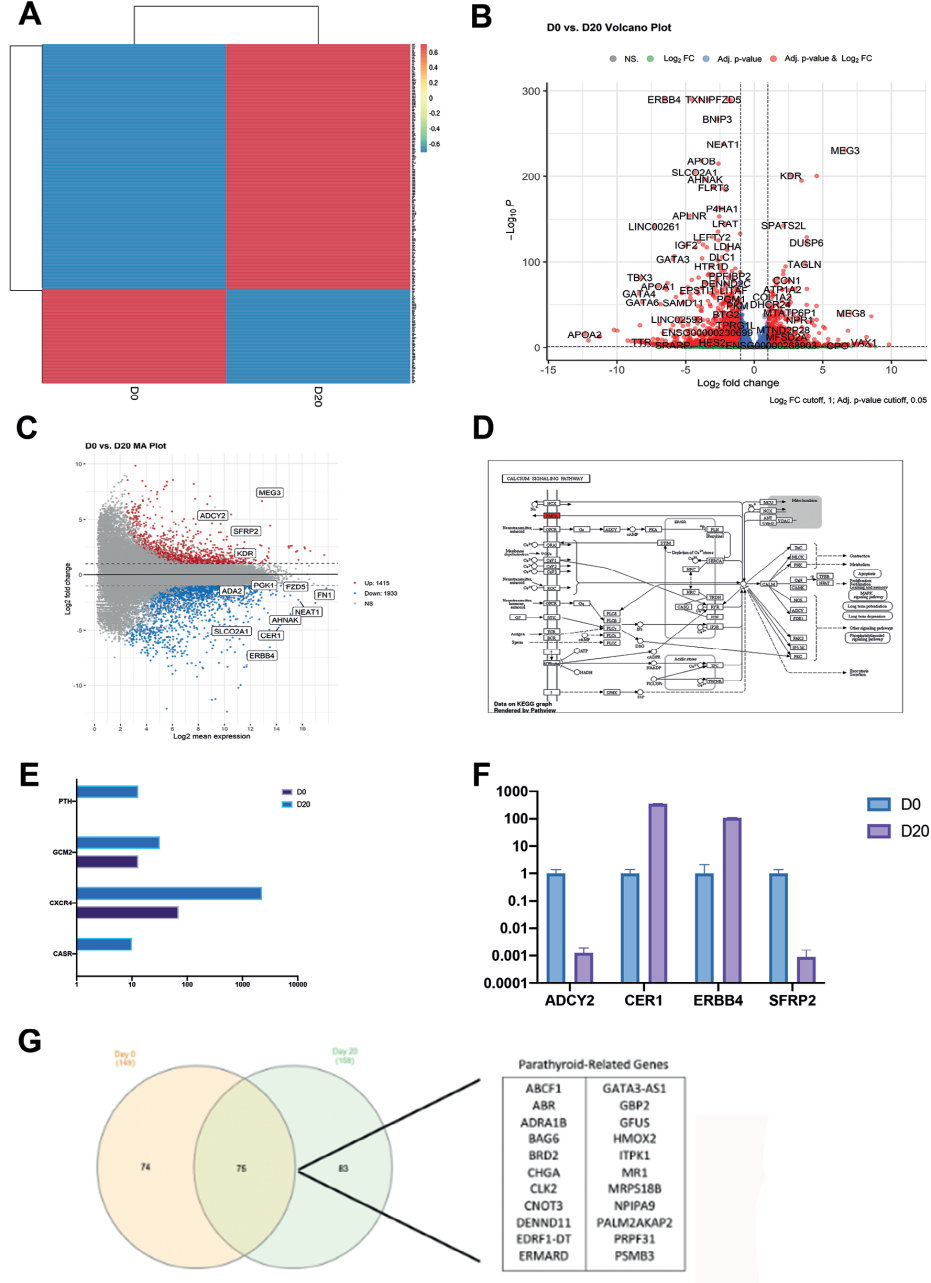
Molecular analysis of parathyroid organoids by RNA‐Seq. A) Gene clustering at Day 0 and Day 20, n = 2. B) Volcano map illustrating the changes from Day 0 to Day 20, n = 2. C) MA plot comparing the data from Day 0 to Day 20, n = 2. D) KEGG pathway focuses on calcium signaling, n = 2. E) Enumeration of parathyroid hormone‐related genes from Day 0 to Day 20. n = 2. F) qPCR analysis to validate the RNA‐Seq results in the Day0‐Day20 experimental groups. n = 2. G) A Venn diagram of the genes related to the parathyroid differentiation at Day0‐Day20 samples, n = 2.

Heatmap cluster analysis of RNA sequencing (RNA‐Seq) showed a distinct separation of control cells (undifferentiated iPSCs at day 0) and parathyroid organoids (at day 20) (**Figure** **4**A). The hierarchical clustering and heat map analysis of the genes that were expressed differently on days 0, 3, 5, 10, 15, and 20 revealed that days 10 and 15, as well as days 3 and 5, formed distinct clusters that were separate from day 0 (Figure S14A, Supporting Information). The Volcano plot (Figure 4B) clearly distinguished the genes that play a significant role in the differentiation process between day 0 and day 20. The MA Plot revealed the presence of several notable genes, such as ADCY2, SFRP2, KDR, and MEG3 (upregulated), as well as ADA2, PGK1, FZD5, FN1, NEAT1, AHNAK, CER1, ERBB4, and SLCO2A1 (downregulated) (Figure 4C). Genes implicated in the calcium signaling pathway were selected from the relevant genes using the KEGG pathway (Figure 4D). Principal component analysis (PCA) demonstrated the proximities between the samples. Day 3 samples were significantly separated from days 0, 5, 10, 15, and 20 (PC1 39%). Days 10 and 20 were also separated by 22.3% variance (Figure S14B, Supporting Information). Additionally, the correlation coefficient matrix supported these data by showing a close correlation between day 0 and day 5 (0.94) and a low correlation between day 15 and day 20 (0.22) and day 10 and day 20 (0.28) (Figure S14C, Supporting Information). The RNA‐Seq analysis revealed that the parathyroid‐related genes PTH, Gcm‐2, CxCr4, and CasR exhibited positive expression. The column graph displays the corresponding gene counts increased on day 20 (Figure 4E). On the 20th day, the gene expression of two upregulated genes, SFRP2 and ADCY2, and two downregulated genes, CER1 and ERBB4, was assessed (Figure 4F). The Venn diagram table (Figure 4G) shows that on day 20, there was an increase in the expression of parathyroid‐related genes including ABCF1, ABR, ADRA1B, BAG6, BRD2, CHGA, CLK2, CNOT3, DENND11, EDRF1‐DT, ERMARD, GATA3‐AS1, GBP2, GFUS, HMOX2, ITPK1, MR1, MRPS18B, NPIPA9, PALM2AKAP2, PRPF31, and PSMB3. The biological process analysis (Figure S15A, Supporting Information) determined that embryonic morphogenesis, circulatory system development, and tube development were all enhanced by differentiation through day 20. Furthermore, there was an observed increase in molecular functions, such as the activity of DNA‐binding transcription factors, cis‐regulatory region sequence‐specific DNA binding, and calcium ion binding (Figure S15B, Supporting Information). Additionally, there was an increase in cellular components, including the extracellular matrix and plasma membrane region (Figure S15C, Supporting Information). Furthermore, KEGG pathway analysis revealed that the TGF and PI3K‐Akt signaling pathways were significantly upregulated (Figure S15D, Supporting Information).

### 2.5 Characterization of iPSC‐Derived Parathyroid Organoids at the Protein Level

**Figure 5:**
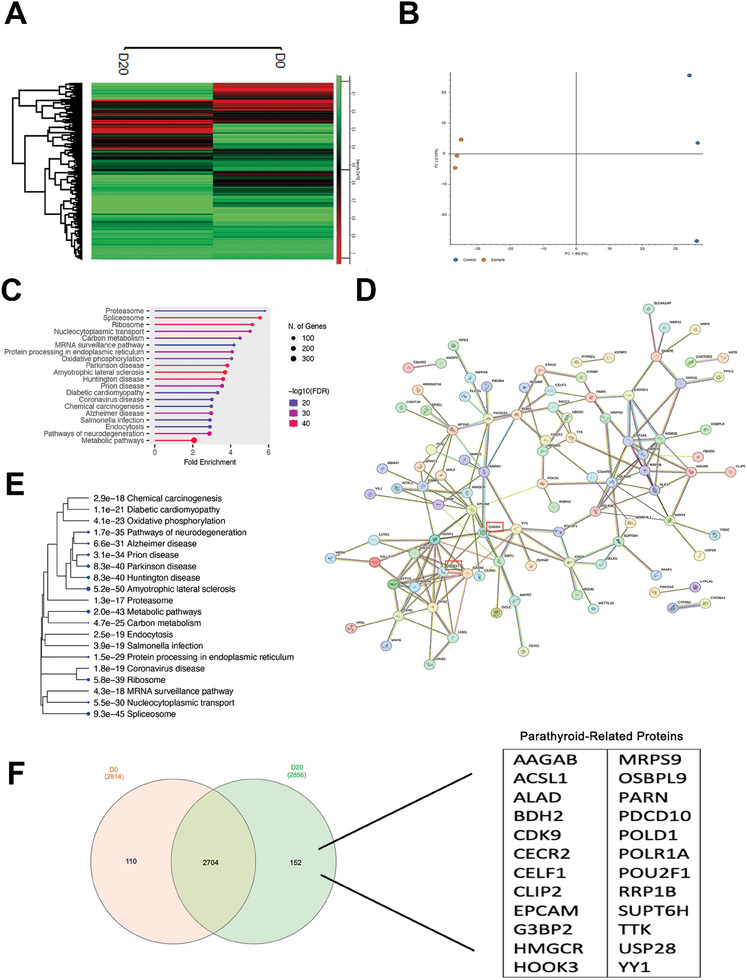
Protein profiling analysis of parathyroid organoids at days 0 and 20. A) Hierarchical clustering with heat map analysis. n = 2. B) PCA for all genes in days 0 and 20. n = 2. C) GO enrichment analyses based on the genes identified in (A) using the lollipop chart. x axis is −Log (FDR), FDR = p value cut‐off = 0,05, n = 2. D) Protein‐protein association networks of days 0 and 20 using the STRING protein association network database for each transcript cluster in (B), n = 2. E) A hierarchical clustering tree summarizing the correlation among significant pathways between day 0 and day 20, n = 2. F) Venn diagram of parathyroid‐related proteins at day 20, n = 2.

The heat map illustrating the findings of the LC‐MS/MS analysis revealed that the protein expression levels of parathyroid organoids on day 20 were different from those of iPSCs on day 0 (**Figure** **5**A). With a 13.5% PCA, the hierarchical cluster analysis revealed that day 20 parathyroid organoids and day 0 iPSCs were distinct (Figure 5B). The lollipop chart demonstrated the identification of the ribosome, metabolic pathways, and spliceosome through Gene ontology (GO) enrichment analyses (Figure 5C). Protein‐protein interactions proved that the endoderm‐based differentiation protocol for Sox‐17 and CxCr4 proteins was effectively accomplished (Figure 5D). A hierarchical clustering tree concisely represents the correlation between significant pathways from day 0 to day 20, suggest the spliceosome and metabolic pathways (Figure 5C,E). Additionally, the Venn diagram demonstrated the presence of parathyroid‐related proteins on day 20 which are CDK9, CECR2, CELF1, CLIP2, EPCAM, G3BP2, HMGCR, HOOK3, MRPS9, OSBPL9, PARN, PDCD10, POLD1, POLR1A, POU2F1, RRP1B, SUPT6H, TTK, USP28, and ACSL1 (Figure 5F). Furthermore, GO analyses were conducted, encompassing protein class, molecular function, and biological process, which were evaluated using Protein Analysis Through Evolutionary Relationships (PANTHER) (Figure S16, Supporting Information). Molecular roles including binding and catalytic activity (Figure S16A, Supporting Information), biologically processes including cellular activities, developmental processes, and biological regulation (Figure S16, Supporting Information), and protein class including transposable element proteins, enzymes involved in metabolite‐interconversion, and enzymes responsible for protein modification (Figure S16C, Supporting Information) were observed in parathyroid organoid.

### 2.6 Activity of Parathyroid Organoids in a Rat Parathyroidectomy Model

**Figure 6:**
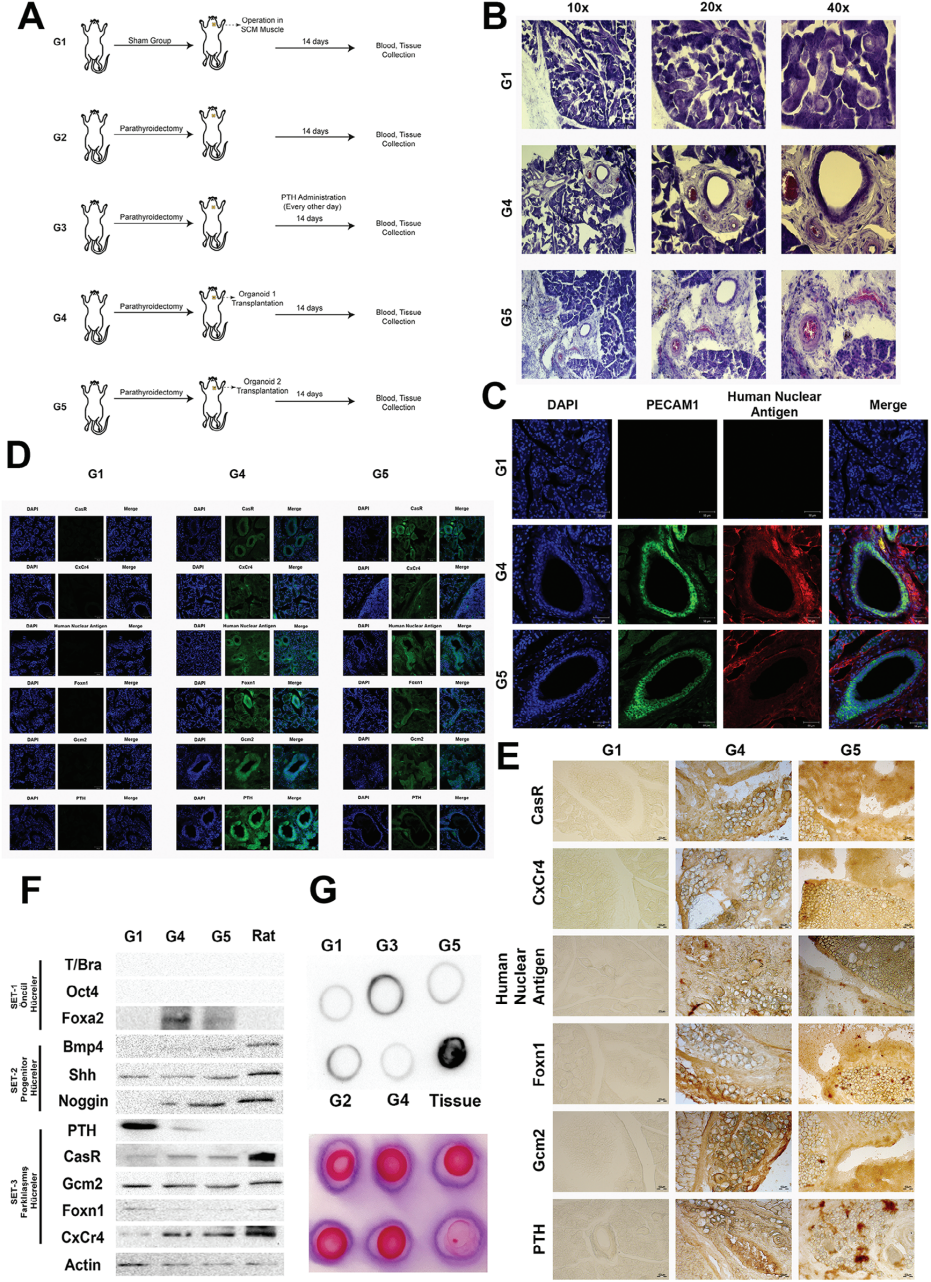
The functional analysis of parathyroid organoids in a parathyroidectomized rat model. A) Schematic illustration of the experimental groups in vivo. B) Histological examination of integration site slices with a thickness of 10 µm. Samples were stained with H&E. Scale bar: 100 µm, 50 µm, 10 µm, n = 3. C) PECAM1 immunofluorescence staining at integration site. Scale bar: 50 µm, n = 3. D) Immunofluorescence staining of human nuclear antigen, CasR, CxCr4, Foxn1, Gcm2, and PTH in the organoids at integration site region. Scale bar: 50 µm, n = 3. E) Human nuclear antigen, CasR, CxCr4, Foxn1, Gcm2, and PTH immunohistochemistry of integration site region. Scale bar: 50 µm, n = 3. F) Western blot analysis of organoids extracted from integration site region. T/Bra, Oct4, Foxa2, Bmp4, Shh, Noggin, PTH, CasR, Gcm2, Foxn1, and Cxcr4 protein expressions were detected. n = 3. G) The concentration of PTH in all in vivo groups’ serum and rat parathyroid tissue, along with the utilization of Ponceau S stain to quantify total protein. n = 3.

The functional activity and tissue integration potential of parathyroid organoids were demonstrated by the transplantation of organoids into parathyroidectomized rats. Two sets of organoids (mature organoids at day 20 and mature organoids that were passaged two times) were compared to control animals (**Figure** **6**A; Table S3, Supporting Information). 5‐ALA injection at different doses (100 mg kg^−1^, 300 mg kg^−1^, and 500 mg kg^−1^) followed by 2 h incubation and visualization under 405 nm xenon light was used for 5‐ALA dose optimization during parathyroid tissue extraction (Figure S17, Supporting Information). 300 mg kg^−1^ 5‐ALA was used during parathyroidectomy and transplantation experiments. There were no significant statistical differences in the weight, food intake, and water consumption rates of the rats for 14 days after organoid transplantation into the sternocleidomastoid (SCM) muscle (Figure S18B‐D, Supporting Information). The concentrations of each mineral (calcium, phosphorus, magnesium, manganese) were analyzed at normal levels following in vivo transplantation (Figure S19, Supporting Information). A notable increase in vascularization was observed in the post‐transplantation integration areas of the groups that received organoids compared to the control group (Figure 6B). Furthermore, it was evident that these sites display morphological characteristics comparable to those observed in the parathyroid organoid within the Matrigel dome (Figure 6B). At the end of 14 days, no sign of rejection was seen in transplanted rats. Furthermore, CD68 staining, which is a marker for macrophages, was conducted. Human nuclear antigen demonstrated the presence of organoids in the sections, while no significant staining was found for CD68 (Figure S20A, Supporting Information). Additionally, IL‐2 expression, an indication of immunological response, did not show significant changes in organoid transplanted groups (Figure S20B, Supporting Information). No significant differences were observed in the compact bone area of the rats during H&E staining compared to the control group (Figure S21, Supporting Information). Upon thoroughly examining H&E staining of the small intestine, crypt structures were observed in all experimental groups (Figure S22, Supporting Information). The number of crypts in the organoid‐transplanted groups was similar to that of the control group. Additionally, it is essential to note that the number of crypts was significantly lower in the parathyroidectomized group and PTH injected groups (G2, G3) (Figure S23, Supporting Information). Moreover, a notable reduction in goblet cells was observed in G2 and G3 (Figure S23, Supporting Information). Post‐transplantation, the structural characteristics of the kidney tissue remained unaltered, similar to the control group. In addition, there were no notable alterations observed in the structure of the Bowman capsule among the groups that received transplanted organoids (Figure S24, Supporting Information). The injection sites obtained after transplantation showed double positive results for human nuclear antigen, indicating the presence of our organoids, and PECAM1, indicating endothelial structures, in the organoid groups compared to the control group (Figure 6C). In addition, there was a significant rise in PTH‐related markers (CasR, CxCr4, Foxn1, Gcm2, and PTH.) in the organoid transplantation groups (G4, G5) compared to the control group (G1) (Figure 6D). Specifically, there were distinct organoid‐like clustered structures that can be observed in the human nuclear antigen staining (Figure 6D). The IHC analyses also confirmed the presence of these structures and positive staining for CasR, CxCr4, Foxn1, Gcm2, PTH, and human nuclear antigen (Figure 6E). CasR, CxCr4, and Gcm2 proteins showed similar levels of expression in rat tissue by western blot analysis (Figure 6F). Upon examining the expression of PTH protein each rats’ serum individually and by pooling the proteins, it was observed that high levels of PTH expression were present in the samples from the group injected with PTH (G3) and group given passaged organoids (G5) (Figure S25, Supporting Information). Passaged organoids had PTH secretion prior to transplantation as mature day 20 organoids (Figure S26, Supporting Information). Furthermore, the measurement of PTH was conducted using dot blot analysis in both pooled experimental groups and rat tissue. No significant disparity was detected when compared to the expression of total protein (as determined by Ponceau S Stain) (Figure 6G; Figure S26, Supporting Information). Additionally, when the expressions of T/Bra, Oct4, Foxa2, Bmp4, Shh, Noggin, PTH, CasR, Gcm2, and Cxcr4 genes in integration groups, it was noted that the passaged organoid transplanted group (G5) exhibited a Gcm‐2 gene expression pattern (Figure S27, Supporting Information).

## 3 Discussion

Hypoparathyroidism, which refers to the loss of parathyroid gland function, is a frequent and enduring consequence that often occurs following thyroid and parathyroid surgery, as well as ablative laryngeal surgery. The incidence of persistent hypoparathyroidism following thyroid surgery ranges from 1% to 15%.^[^ ^15^ ^]^ Hypoparathyroidism results in long‐term low calcium levels in the blood and is a kind of bone disease characterized by reduced bone turnover. The existing techniques for replacing the missing parathyroid hormone are insufficient for effective management of this condition. There is a large population of individuals suffering from this situation. Hypoparathyroidism necessitates administering high doses of calcium and vitamin D.^[^ ^16^ ^]^ Despite being a potential therapy option, allogeneic transplantation of parathyroid cells faces challenges related to the use of immunosuppressants and the rapid degradation of tissue.^[^ ^17^ ^]^ This scenario necessitates long‐term pharmaceutical use for numerous patients.^[^ ^18^ ^]^ The limited proliferative capacity of parathyroid cells impedes their therapeutic application, as they cannot be sustained by prolonged cultivation following isolation from the patient.^[^ ^19^ ^]^ To overcome this limitation, researchers have investigated parathyroid cell differentiation from extracted thymus and tonsil tissue,^[^ ^20^, ^21^ ^]^ as well as ESCs.^[^ ^13^ ^]^ Our approach will enable the generation of 3D parathyroid organoids from iPSCs. iPSCs do not possess the ethical dilemmas associated with ESCs. Due to their pluripotent properties, these cells can be transformed into the specific cell types and can serve as a customized material for cellular therapy.^[^ ^22^ ^]^ Therefore, generation of iPSCs from the patient's somatic cells and differentiating them into organoids could be an important clinical choice for parathyroid transplantation. Parathyroid cells can be derived from ESCs, as demonstrated by Bingham et al. in 2009.^[^ ^13^ ^]^ According to reports, human parathyroid tissues obtained through surgical excision or small needle aspiration can be covered with Matrigel to produce parathyroid organoids from the patient's tissue.^[^ ^23^ ^]^ Furthermore, a recent research has created a parathyroid organoid model by encapsulating human ESC‐derived parathyroid (hESC‐PT) cells in Matrigel. In this protocol, Wang et al. used a 21‐day protocol and showed hormone secretion.^[^ ^14^ ^]^

Biochemical, morphological, histological, and molecular analyses demonstrated that iPSC‐ derived parathyroid organoids could produce hormone and exhibit a structure that resembles the parathyroid endocrine gland. Gcm2 has a crucial role in controlling the development of parathyroid glands, as it is necessary for differentiation and survival.^[^ ^24^, ^25^, ^26^ ^]^ Mice lacking Gcm2 did not develop parathyroid glands and exhibited signs of hypoparathyroidism.^[^ ^27^ ^]^ Simultaneously, Gcm2 attaches to the CaSR promoter and triggers the activation of the CaSR gene, thereby facilitating the development of parathyroid glands.^[^ ^28^ ^]^ The results of our study demonstrated the presence of Gcm2 and CasR in parathyroid organoids, as evidenced by both morphological (Figure 3A) and molecular (Figure 4E) analysis. We performed detailed molecular analysis using RNA‐Seq to check marker gene transcriptome of organoids. Both RNA‐Seq and LC‐MS/MS analyses showed the successful differentiation of parathyroid organoids. Responses to calcium administration and Fluo‐4 dye were observed in our parathyroid organoids by decreasing PTH production and increasing Fluo‐4 transmission respectively. Literature reports indicate that cinacalcet, a calcimimetic drug, decreases serum calcium and phosphorus levels.^[^ ^29^ ^]^ In our study, administering cinacalcet in the presence of 4 mM calcium resulted in a reduction in calcium and phosphorus levels secreted to the culture medium, which indicates the response of parathyroid organoids to the drugs. Ritter et al. separated bovine parathyroid tissue cells and generate a pseudogland structure in a collagen matrix environment which produce PTH at day twenty in response to calcium. Our organoids also responded to calcium in vitro by upregulating PTH expression. These results are in consistent with the literature as, elevated extracellular calcium levels during regular physiological processes also inhibit the secretion of PTH.^[^ ^23^ ^]^ In addition, parathyroid organoid were able to be labelled by 5‐ALA, which induces red fluorescent in response to 405 nm in vivo.^[^ ^30^ ^]^

In a previously published research, parathyroid‐like cells were derived from tonsil‐derived mesenchymal stem cells (TMSCs) and encapsulated in 3D alginate microbeads for the treatment of hypoparathyroidism.^[^ ^31^ ^]^ PTH staining was much less obvious in differentiated cell transplanted group. We also observed PTH release 14 days after transplantation. Both literature and our research indicated that organoid transplantation can be successful in terms of tissue integration and hormone release. Althuogh contemporary surgical transplantation in hypoparathyroidism cases extensively use microencapsulation technology,^[^ ^32^, ^33^ ^]^ we use Matrigel as a matrix material to embed our parathyroid organoids for in vivo transplantation. It might be necessary to do further experiments using microencapsulation technology or alternative matrix materials in the future. Tonsil cells were differentiated from parathyroid cells in another study and transplanted to the rats with a Matrigel matrix subcutaneously. In this study, parathyroid hormone release were detected until the twenty‐eighth day.^[^ ^20^ ^]^ Although we used iPSC‐derived parathyroid organoids and preferred intramuscular transplantation and measured hormone levels at the end of 14 days, both studies showed similar results in terms of successful hormone production.

Although tissue originated and ESC derived parathyroid cell differentiation protocols exists in the literature, there is no iPSC‐derived parathyroid organoid generation protocol. The most recent published study generated parathyroid‐like cells from ESCs and formed parathyroid organoid.^[^ ^14^ ^]^ We generated iPSC‐derived 3D parathyroid organoids and extensively characterized their functional properties in in vitro and in vivo. Getting 3D organoid structures from pluripotent stem cells that can show how the parathyroid organ works is very important for scientists studying molecular biology, developmental biology, genetic and chronic disease models, hormone production, and personalized drug and treatment research. This is particularly relevant to the study of parathyroid tissue in the fields of anatomy, physiology, and endocrinology. Consequently, this investigation will yield significant and original research data for the field. This article lays the groundwork for parathyroid organoids transplantation and hypoparathyroidism treatment use. This protocol might open a new field for personalized drug response analysis. Long term hormone production, storage, morphological and functional characteristics of parathyroid organoid culture should be studied for further research and potential applications.

This study demonstrated the potential of iPSC‐derived parathyroid organoid in a parathyroidectomized rat model. However, the possible functional activity should be studied in different experimental models which mimics actual physiological diseases, conditions related to parathyroid hormone function. Long term transplantation and tissue integrity, long term in in vivo hormone release in response to increasing and decreasing Ca in the bloodstream and the utilization of alternative matrix materials should be studied prior to further clinical investigations.

## 4 Experimental Section

### Cellular Reprogramming

Human dermal fibroblast cells (HDFCs, CC‐2511, Lonza, Lot no: 000 0670494) were cultivated in a humidified chamber at 37 °C in complete fibroblast medium^[^ ^34^ ^]^ (Table S1, Supporting Information). HDFCs at passage seven were reprogrammed into iPSCs using the StemRNA 3rd Gen Reprogramming Kit (ReproCELL, USA).^[^ ^35^ ^]^ Briefly, HDFCs were seeded onto hESC‐qualified Matrigel (Corning, USA) coated 12‐well plate (TPP, Switzerland) in a complete fibroblast medium. Cells were transfected with Oct4, Sox2, Klf4, c‐Myc, Nanog, and Lin28 reprogramming RNAs for three consecutive days using Lipofectamine RNAiMAX transfection reagent (Invitrogen, UK) and OptiMEM (Gibco, USA). Colonies were manually selected for ALP expression (ALP+) after 10 days and cultured in mTESR Plus medium (StemCell Technologies, USA) according to the feeder‐free protocol.^[^ ^34^ ^]^

### Live ALP Assay

iPSCs were stained with an ALP live stain kit (ThermoFisher Scientific, USA) according to the manufacturer's instructions.^[^ ^36^ ^]^ Briefly, cells were washed with fresh, pre‐warmed DMEM/F‐12 media (Gibco, USA). 500X stock solutions were diluted in DMEM/F‐12 media and applied to the adherent iPSCs. iPSCs were incubated with dye solution for 30 min and then washed twice with DMEM/F‐12 media to remove excess dye. After the final wash, iPSCs were visualized under a fluorescent microscope (Axio Vert.A1; Zeiss, Germany). Images were captured 30–90 minutes after staining, and positive colonies were selected.

### Trilineage Differentiation

iPSCs were differentiated into endoderm, ectoderm, and mesoderm lineages according to STEMdiff™ Trilineage Differentiation Kit protocol (STEMCELL Technologies, USA).^[^ ^37^ ^]^ Briefly, cells were seeded onto hESC‐qualified Matrigel‐coated 12‐well plates and treated with endoderm and mesoderm media for 5 days and ectoderm media for 7 days. After differentiation, cells were prepared for further characterization experiments, including Pax6, Sox17, and T/Bra immunostaining and quantitative polymerase chain reaction (qPCR) analysis.

### Pluripotency Marker Expression

iPSCs were stained with Nanog, Oct3/4, TRA‐1, and SSEA‐1 antibodies for pluripotency analysis.^[^ ^38^ ^]^ Cells were fixed with 4% PFA (ThermoFisher Scientific, USA) and permeabilized with a 0.1% Triton‐X 100 (Sigma‐Aldrich, UK) solution. Afterwards, cells were washed with PBS (Gibco, USA) and treated with 1% BSA (Sigma‐Aldrich, UK) for blocking. Cells were incubated with the following primary antibodies: Nanog, Oct3/4, TRA‐1, and SSEA‐1 (SantaCruz Biotechnology, USA) overnight at 4 °C. Cells were rinsed and incubated with secondary antibodies including anti‐mouse AlexaFluor 488 and anti‐rabbit AlexaFluor 488 (Invitrogen, USA) for 30 min at room temperature. DAPI (1:1000) (ThermoFisher Scientific, USA) was used for nuclei staining. The images were taken using a confocal microscope (LSM 700; Zeiss, Heidelberg, Germany).

### Chromosomal Abnormalities by qPCR

Genomic DNA was isolated using the High Pure Genomic DNA Purification Kit (Roche, USA). qPCR‐based hPSC Genetic Analysis Kit (STEMCELL Technologies, USA) was used to detect karyotyping abnormalities in iPSCs according to the manufacturer's instructions.^[^ ^39^ ^]^ Chr4p was used as a control chromosomal region.

### Telomerase Activity of iPSCs

Telomerase activity quantification qPCR assay kit (ScienCell, USA) was used to detect telomerase activity according to the manufacturer's protocol.^[^ ^40^ ^]^ qPCR was performed using the CFX96 RT‐PCR system (Bio‐Rad, USA) to assess telomerase activity. The relative telomerase activity was calculated by the GraphPad Prism 9 (GraphPad Software, SanDiego, California, USA, www.graphpad.com).

### Generation of Parathyroid Organoids from iPSCs

The differentiation of iPSCs into parathyroid organoids was achieved by adapting Bingham's approach, as described in Table S1 (Supporting Information).^[^ ^13^ ^]^ The process of differentiation continued for 20 days. During the initial 10‐day period, the N2B27 culture medium was supplemented with 100 ng mL^−1^ of Activin A and 10 µM of Y‐27632. During the remaining 10 days, 50 ng mL^−1^ Shh was introduced into the culture medium. On the fifth day of the differentiation process, 30 µL organoid domes were formed using a 1:1 mixture of 15 µL differentiation media and 15 µL Matrigel. The dome culture was incubated at 37 °C for 30 minutes for polymerization. Parathyroid organoids were characterized using morphological examination, RNA‐Seq, LC‐MS/MS, inductively coupled plasma mass spectrometry (ICP‐MS), qPCR, western blot WB, and immunofluorescence techniques on day 20.

For the subculturing of parathyroid organoids, Matrigel domes were dissolved with cold PBS and centrifuged at 300xg for 3 min. Following centrifugation, the cells were mixed with 15 µL of differentiation medium and 15 µL of Matrigel. The dome structure was initiated and incubated at 37 °C for 30 min. Then, a differentiation culture medium was added. The passage procedure can be repeated every 2–3 days based on the confluency.

### Morphological Image Analysis of Parathyroid Organoids

Parathyroid organoids were visualized at various focuses, from the outermost to the innermost cell layer, using an inverted microscope (Nikon, Japan) equipped with TE200 camera and NIS Elements BR 2.30 software (Nikon, Japan) at the specified time points (Days 0, 3, 5, 10, 15, and 20). The morphological changes of the organoids were monitored by quantifying the crypt‐like structure, area, and size of the organoids by Image J (NIH, Bethesda, USA).^[^ ^41^ ^]^ Statistical analyses were performed using GraphPad Prism 9 software (GraphPad Software, San Diego, CA, USA). Stereomicroscope (Zeiss, Oberkochen, Germany) and ZEN 3.2 Blue software (Zeiss, Germany) were utilized to examine the intricate 3D arrangement of parathyroid organoids meticulously.

### PTH Secretion

Dot Blot analysis was performed to determine the PTH concentration in cell culture secretome and animal serum samples. Total proteins were isolated from the samples (in vitro and in vivo samples) (Table S2, Supporting Information) using RIPA Buffer (Santa Cruz Biotechnology, USA). A volume of 2 µL (0.2 µg mL^−1^) from each sample was introduced onto the Nitrocellulose membrane (Bio‐Rad, USA). After absorption, the membrane was blocked using 5% skimmed milk. The PTH primary antibody was then added to the samples and subjected to overnight incubation at 4 °C. Following incubation, the membranes were washed three times with TBS‐T. Subsequently, a secondary antibody was introduced and incubated for 1 h at room temperature. Following the incubation period, the membranes underwent three washes with TBS‐T analyzed using the luminometer system (Bio‐Rad, USA). Next, the membranes were subjected to staining using Ponceau S Staining solution (ThermoScientific, USA) to determine the total protein content.

### Osteogenic Differentiation Analysis

MC3T3‐E1 (ATCC, CRL‐2593) mouse preosteoblast cells were used as a model cell line to demonstrate in vitro osteogenic differentiation activity.^[^ ^42^ ^]^ Cells were seeded in a 12‐well plate at a cell density of 10 × 10^4^ cells. During the in vitro differentiation experiments, 20% of the conditioned media collected from organoids (days 0, 3, 5, 10, 15) were mixed with osteogenic differentiation medium and then administered to MC3T3‐E1 cells for 14 days. After differentiation, Alizarin Red staining, gene expression analysis of Runx, Col I, and Osteonectin genes, and immunocytochemistry analysis of Osteocalcin and Collagen type I were conducted. A standard cell culture medium was used as the negative control group. A cell culture medium supplemented with parathyroid hormone (Invitrogen, USA, 10 nM) was used as the positive control group.

### Calcium Response Analysis of Parathyroid Organoids

Organoids were subjected to different calcium concentrations (0, 2, and 4 mM) for two time points (30 minutes and 1 hour) after the 20‐day differentiation protocol. Calcium concentration in the cell culture media was subsequently quantified using an ICP‐MS device (Thermo, Xseries 2), as described in Section 19.2 to investigate the calcium response. p‐ERK and PTH levels of organoids were checked by Western blot in response to calcium.

Cinacalcet (Cineset, 30 mg, Nobel İlaç) which is a calcimimetic agent and allosteric modulator of CasR was dissolved in distilled water as stock solution (2.5 mM). 50 nM cinacalcet^[^ ^43^ ^]^ was applied to parathyroid organoids in a culture medium which contains 2 mM Ca and 4 mM Ca for 1 hour. After administration, serum Ca and P measurements were performed by ICP‐MS, Viable cell number was calculated by trypan blue staining and cell counting.

### RNA Sequencing (RNA Seq) Analysis

Total RNA was extracted from organoid samples at different time points (Table S2, Supporting Information) using RNeasy plus mini kit (Qiagen, Germany). RNA concentration and quality were estimated using a Nanodrop 2000 Spectrophotometer (Thermo Scientific, USA). Samples with good RIN values (7.0–8.5) were used for cDNA conversion and paired‐end library preparation using the Illumina® Stranded Total RNA Prep, Ligation with Ribo‐Zero Plus (Illumina, USA), according to the manufacturer's instructions. The library quality was assessed using the Bioanalyzer (Agilent Technologies, CA, USA). Then, the libraries were sequenced using the Illumina Novaseq 6000 system and generated 150 bp paired‐end raw reads. The FastQC program was utilized to perform the quality assessment (https://www.bioinformatics.babraham.ac.uk/projects/fastqc). After aligning RNA‐seq reads with STAR to GRCh38 using the default parameters,^[^ ^44^ ^]^ Cufflinks was utilized to assemble the reads.^[^ ^45^ ^]^ Fragment abundance per kb of exon per million fragments mapped (FPKM) was utilized to quantify transcript abundance. Differentially expressed genes (DEGs) were identified using Cuffdiff^[^ ^45^ ^]^ when the adj. p‐value was less than 0.1 and the FPKM was greater than 2. ShinyGO was utilized to enrich the Gene Ontology and KEGG pathways.^[^ ^46^ ^]^ The correlation matrix analysis and generation of volcano diagrams were conducted using GraphPad Prism 9 (GraphPad Software, SanDiego, California USA, www.graphpad.com). Principal component analysis (PCA) and hierarchical clustering with heat maps were constructed using the ClustVis online tool^[^ ^47^ ^]^ (https://biit.cs.ut.ee/clustvis/). A Venn diagram was generated utilizing the InteractiVenn online utility.^[^ ^48^ ^]^ Enhancement analysis functional profiling was conducted using the g:Profiler website (https://biit.cs.ut.ee/gprofiler/) by Raudvere et al. (2019).^[^ ^49^ ^]^

### LC‐MS/MS Analysis

The protein profile of the cells at day 0 (iPSCs) and day 20 (parathyroid organoids) was assessed using liquid chromatography/mass spectrometry (LC‐MS/MS) analysis, using the established technique as described previously.^[^ ^50^ ^]^ In this study, samples were taken at two‐time points during the differentiation process of parathyroid organoids: day 0 and day 20. Following this, the proteins derived from these samples were sent for examination using SDS‐PAGE. The gel was immobilized in a solution of 40% methanol, 10% acetic acid, and 50% water and subsequently treated with colloidal Coomassie Blue G‐250 for staining purposes. After performing in‐gel tryptic digestion on the excised protein bands, the resulting peptides were subjected to analysis using a Dionex Ultimate 3000 RSCL nanosystem (Thermo Scientific, USA) linked to a Q Exactive mass spectrometer (Thermo Fisher Scientific, USA) through LC‐MS/MS. The Xcalibur 4.0 software, developed by Thermo Fisher Scientific in the United States, was utilized in this study. The experimental procedure involved the utilization of High‐Performance Liquid Chromatography (HPLC) with mobile phases A and B, followed by the acquisition of full‐scan MS spectra. Following determining the top ten precursor ions for MS/MS, protein identification analysis was conducted utilizing Proteome Discoverer 2.2 software, developed by Thermo Scientific in the United States. The study involved searching against the Uniprot/Swissprot database. ShinyGO was utilized to enrich Gene Ontology and KEGG pathways.^[^ ^46^ ^]^ DisplayR (Displayr Australia Pty Ltd) examined correlation matrixes. Using the ClustVis online tool (https://biit.cs.ut.ee/clustvis/),^[^ ^47^ ^]^ hierarchical clustering with heat map and principal component analysis (PCA) were generated. InteractiVenn was used to create a Venn diagram.^[^ ^48^ ^]^ The g:Profiler website (https://biit.cs.ut.ee/gprofiler/) was used to execute functional profiling of enrichment analysis.^[^ ^49^ ^]^ Using the Perseus software package (version: 1.5.5.3) (https://maxquant.net/perseus/),^[^ ^51^ ^]^ hierarchical clustering with heat map, protein distribution histogram, and profile plot were generated for protein profiling with LC/MS. The STRING database (https://string‐db.org) was used to represent the network of protein interactions.^[^ ^52^ ^]^ For gene ontology and pathway analysis of LC/MS data, the PANTHER (version: 17.0) online tool (http://www.pantherdb.org) was used.^[^ ^53^, ^54^, ^55^ ^]^

### Organoid Transplantation In Vivo

Parathyroid organoids were transplanted into parathyroidectomized rats to determine the in vivo functional activity of parathyroid organoids and integration potential in the transplantation area. The following procedure has been approved by the İnönü University, Faculty of Medicine, Experimental Animals Laboratories (Approval number/date:2019/A‐51, 23.10.2019). Animals were housed at a constant temperature (23 ± 1 °C) and humidity (60 ± 10%), maintained at a 12‐hour light/dark cycle, and fed ad libitum. A total of 40 male rats were divided into 5 groups, with 8 rats in each group (Table S3, Supporting Information). In order to make parathyroid tissues visible for parathyroidectomy, intra‐abdominal injections of 5‐aminolevulinic acid hydrochloride (5‐ALA, Sigma‐Aldrich, UK) were administered at dosages of 100, 300, and 500 mg kg^−1^ and animals were kept at dark for 2 h. A 405 nm wavelength light source was used to visualize parathyroid tissues, and parathyroid glands were removed. Organoids were transplanted inside the SCM muscle. The organoids were embedded in fresh Matrigel (40 µL) at 4 °C, incubated for 15 min at 37 °C, and subsequently introduced into the muscle tissue in a polymerized form. Following the placement of the organoids in the SCM area, a suture was applied internally to the muscle. Subsequently, following the transplantation of the organoid, the area was sutured using silk thread (No. 5.0) and the wound was sterilized by administering a betadine solution (5% Sterile Ophthalmic Prep Solution, Woodstock, IL). In the control group, the SCM muscle region was incised and subsequently sutured. The third group received intraperitoneal injections of parathyroid hormone daily at a dosage of “0.528 µg per 100 g body weight”. This group was assigned as the positive control group. The weight, food, and water consumption of the animals were assessed during the 14 days. Animals were sacrificed at the end of the 14 days and the target organs including the kidney, small intestine, bone, and transplantation site (muscle tissue), were collected for subsequent analysis.

### PTH Serum Level Measurement

Blood samples were collected from each rat at the end of the animal experiments (14‐day period). The concentration of PTH protein was assessed by Western Blot and Dot Blot analysis. In this study, protein isolation was conducted individually for each rat as well as for the pooled group. Briefly, a volume of 2 µL from each sample was introduced onto the Nitrocellulose membrane and then PTH primary antibody was used to determine PTH level. Also, the membranes were subjected to staining using Ponceau S stain to determine the total protein content.

### Mineral Analysis

The amounts of serum calcium, phosphorus, manganese, and magnesium were determined using an iCAP RQ ICP‐MS device (Thermo Scientific, USA), as documented in the existing literature.^[^ ^56^ ^]^ 100 µL of serum‐separated blood samples were put into the tube. Subsequently, the solution was diluted by adding ultrapure water until a final volume of 5 mL was achieved. The mineral concentrations in each diluted samples were determined using an ICP‐MS apparatus. The device was operated with the following parameters: nebulizer gas flow rate of 1.14 L min^−1^, cool gas flow rate of 14 L min^−1^, power setting of 1051 W, and auxiliary gas flow rate of 0.8 L min^−1^. The experiment included different concentrations of standard solutions (1.11355.0100, Sigma Aldrich, UK).

### Rat IL‐2 ELISA Assay

Collected and pooled serum samples from each rat were analyzed by using Rat IL‐2 ELISA assay (#BMS634, Invitrogen, USA) according to manufacturer's instructions. Briefly, strips were washed with wash buffer. Then, IL‐2 standards and serum samples were added to wells. Biotin‐conjugate was added onto all wells and incubated at room temperature for 3 h on a microplate shaker. Wells were washed with wash buffer 4 times. Then, Streptavidin‐HRP was added to all wells and incubated at room temperature for 1 hour on a microplate shaker. After, wells were washed with wash buffer 4 times. TMB Substrate Solution was added to all wells and incubated avoiding direct light at room temperature for 10 min on a microplate shaker. Finally, stop solution was quickly added to all wells and strips were measured at 450 nm using spectrophotometer.

### Organoid Tissue Integration Analysis

At the end of the 14th day, the transplanted organoids were extracted from the adjacent muscle tissue and subjected to morphological structure analysis, integration with the tissue, and parathyroid marker expression at the gene and protein level. Organoids were subjected to immunocytochemistry analysis, histopathological staining, and gene and protein expression analysis. The organoids were assessed for their parathyroid phenotype using immunostaining with antibodies specific to Foxn1, Gcm2, CasR, PTH, and CxCR4, indicating their complete differentiation. Immunostaining of human nuclear antigen was conducted to visualize the organoids derived from human cells transplanted into rats following parathyroidectomy. Also, immunostaining of PECAM1 was performed to visualize the endothelial fate of organoids. Histopathological investigations were conducted to show tissue morphology using H&E staining (Abcam, UK).^[^ ^57^ ^]^ The samples extracted from integration site of each group have been stored for qPCR and Western blot analysis.

### Bone, Kidney, and Intestinal Tissue Histopathological Analysis

The small intestine and kidney tissues were fixed in a 4% paraformaldehyde (PFA) solution for 2 h at room temperature. Tissues were incubated with fresh 30% sucrose solution overnight at 4 °C. The following day, tissues were frozen‐embedded in disposable molds (Sigma‐Aldrich, UK) and sectioned at a thickness of 10 µm using a cryostat. Tissue morphology was examined with H&E staining using a Zeiss PrimoVert light microscope equipped with an AxioCam ERc5 camera. Intestinal tissue crypt structures and goblet cells were quantified to identify any aberrations in the morphology of normal tissue.

The bone tissues were fixed in 4% PFA solution for 2 h at room temperature, after that, they were decalcified using a 400 mM EDTA solution at 4 °C for 2 weeks. Then, the tissues were incubated with fresh 30% sucrose solution at 4 °C for 16 h. Bone tissues were embedded in disposable molds with OCT solution and then sectioned at a thickness of 10 µm using cryostat. Sections were stained with H&E staining for tissue morphology by Zeiss PrimoVert light microscope equipped with an AxioCam ERc5 camera connection.

### Immunohistochemistry Analysis

The transplantation area (integration site‐muscle tissue) was obtained at the end of animal experiments. The tissues were incubated with a 4% PFA solution for 2 h at room temperature. Subsequently, it was subjected to incubation with a freshly prepared 30% sucrose solution at 4 °C overnight. Then, the tissues were embedded in an OCT solution and sectioned at 10 µm in thickness using a cryostat. The immunohistochemical investigation utilized Foxn1, Gcm2, CasR, PTH, CxCR4, PECAM1, and human nuclear antigen antibodies (Table S5, Supporting Information). Briefly, the sections were fixed with cold acetone for 5 min. Next, the sections were dipped in distilled water five times. The sections were blocked using a 2.5% solution of normal horse serum for 10 min. Subsequently, the sections were incubated with the specified primary antibodies for 1 hour at room temperature. The sections were washed with PBS for 5 min. Subsequently, they were incubated with biotinylated secondary antibodies for 10 min at room temperature. Following the rinsing with PBS, the sections were incubated with streptavidin for 5 min at room temperature. Following a 5‐minute wash with PBS, the sections were incubated with a combination of DAB and DAB substrate (Cell Signalling Technology, USA) for 15 to 30 s. The sections were covered with coverslips using Aqua‐Poly/Mount (Polysciences, USA), a mounting medium. The sections were analyzed using Zeiss PrimoVert light microscope equipped with an AxioCam ERc5 camera connection.^[^ ^58^ ^]^

### PCR Analysis

T/Bra, Foxa2, Oct4, Shh, BMP4, Noggin, CxCr4, CasR, Foxa1, Foxn1, PTH, Gcm2 and Sox17, ADCY2, CER1, ERBB4, SFRP2, AFP, ESRG, FN1, PGK1 (Table S4, Supporting Information) primers were designed using Primer‐BLAST software from the National Center for Biotechnology (Bethesda, MD, USA) and synthesized by Sentegen (Ankara, Turkey). β‐Actin was used as a housekeeping gene. The extraction of total RNA was performed on the samples (in vitro and in vivo samples) listed in Table S2 (Supporting Information). Total RNAs were isolated using RNeasy plus mini kit (Qiagen, Germany) following the manufacturer's instructions. cDNA was synthesized using an iScprit cDNA synthesis kit (Bio‐Rad, USA). SYBR Green (Applied Biosystems, USA) method was used as described previously for qPCR analysis.^[^ ^59^ ^]^ All RT‐PCR experiments were conducted using the CFX96 RT‐PCR system (Bio‐Rad, USA).

### Western Blot Analysis

Total proteins were isolated from the samples (in vitro and in vivo samples) listed in Table S2 (Supporting Information) using RIPA Buffer (SantaCruz Biotechnology, USA). Total protein concentrations were determined using a BCA Protein Assay Kit (Thermo Scientific, USA).^[^ ^59^ ^]^ Protein samples were loaded to the Mini‐PROTEAN TGX Precast Gels (BioRad, USA) at 30 µg per lane and transferred to PVDF membranes (BioRad, USA). Then, membranes were incubated with a blocking solution containing 5% skimmed milk prepared in TBS‐T. After blocking, membranes were incubated overnight at 4 °C with the fallowing primary antibodies: anti‐T/Bra, anti‐Foxa2, anti‐Oct3/4, anti‐Shh, anti‐BMP4, anti‐Noggin, anti‐Foxn1, anti‐Gcm2, anti‐CasR, anti‐PTH, and anti‐CxCr4, anti‐Erk, and anti‐p‐Erk. After washing with TBS‐T three times, membranes were incubated with secondary antibodies (HRP‐conjugated) prepared in a blocking buffer for 1 h. β‐actin antibodies (dilution 1:1000, Cell Signalling, USA) were used as an internal control, and images were taken using the luminometer system (Biorad, USA). Band intensities were quantified by Image Lab software (BioRad, Hercules, CA, USA) and normalized to the β‐actin.

### Immunostaining Analysis

The in vitro organoids at days 0, 3, 5, 10, 15, and 20 and in vivo transplantedorganoids from integration sites were fixed with 4% PFA. After fixation, organoids and tissues were washed with PBS and frozen by embedding in an OCT solution. Organoids were sectioned (10 µm sections) with a cryostat device (SLEE MEV +, Germany). The organoids and tissues were permeabilized in a PBS solution containing 0.1% Triton‐X 100. Then, organoids and tissues were rinsed with PBS and blocked with 1% BSA. Organoids and tissues were incubated with the primary antibodies: T/Bra, Foxa2, Oct4, Shh, BMP4, Noggin, Foxn1, Gcm2, CasR, PTH, CxCr4, PECAM1, Human Nuclear Antigen, CD68 (Table S5, Supporting Information) overnight at 4 °C. Organoids and tissues were rinsed and incubated with secondary antibodies including AlexaFluor 488, AlexaFluor 647 and DAPI (Thermo Scientific, USA) for 45 minutes at room temperature. The images were taken with a confocal microscope (LSM 700; Zeiss, Heidelberg, Germany), and intensities were calculated with ZEN 3.2 Blue software (Zeiss, Germany).^[^ ^60^ ^]^

### Electron Microscopy Analysis

Parathyroid organoids were fixed for TEM, and SEManalysis.

Parathyroid organoids were fixed with 2.5% glutaraldehyde solution (Sigma‐Aldrich, UK) and formaldehyde in PBS for 2 h at room temperature for TEM analysis. After rinsing with dH_2_O, the post‐fixation was done with 1% osmium tetroxide (abcr GmbH, Germany) for 1 hour at fume. The sample was rinsed with PBS five times for 10 min. Then, the sample was dehydrated with 50, 70, 90, and 100% ethanol for 5 min on a stir. Next, the sample was embedded with the Epoxy‐Embedding Kit (Sigma Aldrich, UK). After that, the sample was sectioned with a thickness of 100 nm using an ultramicrotome (EM UC7, Leica, USA). Then, the samples were stained with 2% uranyl acetate altetnative (Ted Pella, USA) for negative staining. Parathyroid organoids were visualized in TEM (JEM‐2100Plus, JEOL, Tokyo, Japan) at 200 kV.^[^ ^61^ ^]^

Parathyroid organoids were fixed with 2% glutaraldehyde prepared in 0.1 M sodium cacodylate buffer (Sigma Aldrich, UK) at 4 °C for 1 h. Fixed samples were washed with PBS and dehydrated with 60, 70, 90, and 100% ethanol series. The dried samples were coated with 2.5 nm thick gold‐platinum (Au/Pt) at 0.08 nm per second with EM ACE200 (Leica, USA) for 30 s, and then SEM images were taken using Zeiss Evo40 (Zeiss, Heidelberg, Germany).^[^ ^62^ ^]^

### AFM Analysis

Parathyroid organoids were fixed with 2% glutaraldehyde prepared in 0.1 M sodium cacodylate buffer at 4 °C for 1 h. Then, the sample was washed twice with deionized water. The sample was placed on a carbon disc and dried at room temperature. Sample surface topographies were analyzed using an XE‐100 AFM system (Park Systems, Korea) equipped with a NSC36B silicon cantilever in contact mode. Scan fields of 40 × 40 µm^2^ on all surfaces were processed using the XEI software (1.18).^[^ ^62^ ^]^

### Statistical Analysis

The data was analyzed using a one‐way analysis of variance (ANOVA) and unpaired t‐test statistical analysis. Statistical analyses were performed using the GraphPad Prism 9 (GraphPad Software, San Diego, CA, USA) program. Unless otherwise indicated in the Figure Legends, organoids were considered biological replicates. The data is presented as the mean ± S.E.M and mean ± SD. Results were statistically significant if the p‐value was less than or equal to 0.05, as asterisks denote. Organoid samples were randomly selected from the culture for experiments and analyses.

### Ethics Approval Statement

All animal experiments have been approved by the İnönü University, Faculty of Medicine, Experimental Animals Laboratories (Approval number/date:2019/A‐51, 23.10.2019).

## Conflict of Interest

The authors declare no conflict of interest.

## Supporting information



Supporting Information

Supporting Information

## Data Availability

The data that support the findings of this study are available from the corresponding author upon reasonable request.
