# Supplementary material for: Induced Pluripotent Stem Cell‐Derived Parathyroid Organoids Resemble Parathyroid Morphology and Function
Source: Adv Sci (Weinh). 2024 Sep 27;11(43):2407567. doi: 10.1002/advs.202407567 (PMC11578294; doi:10.1002/advs.202407567)
Supplement: Supplementary file 1 — Supporting Information [file ADVS-11-2407567-s002.docx]

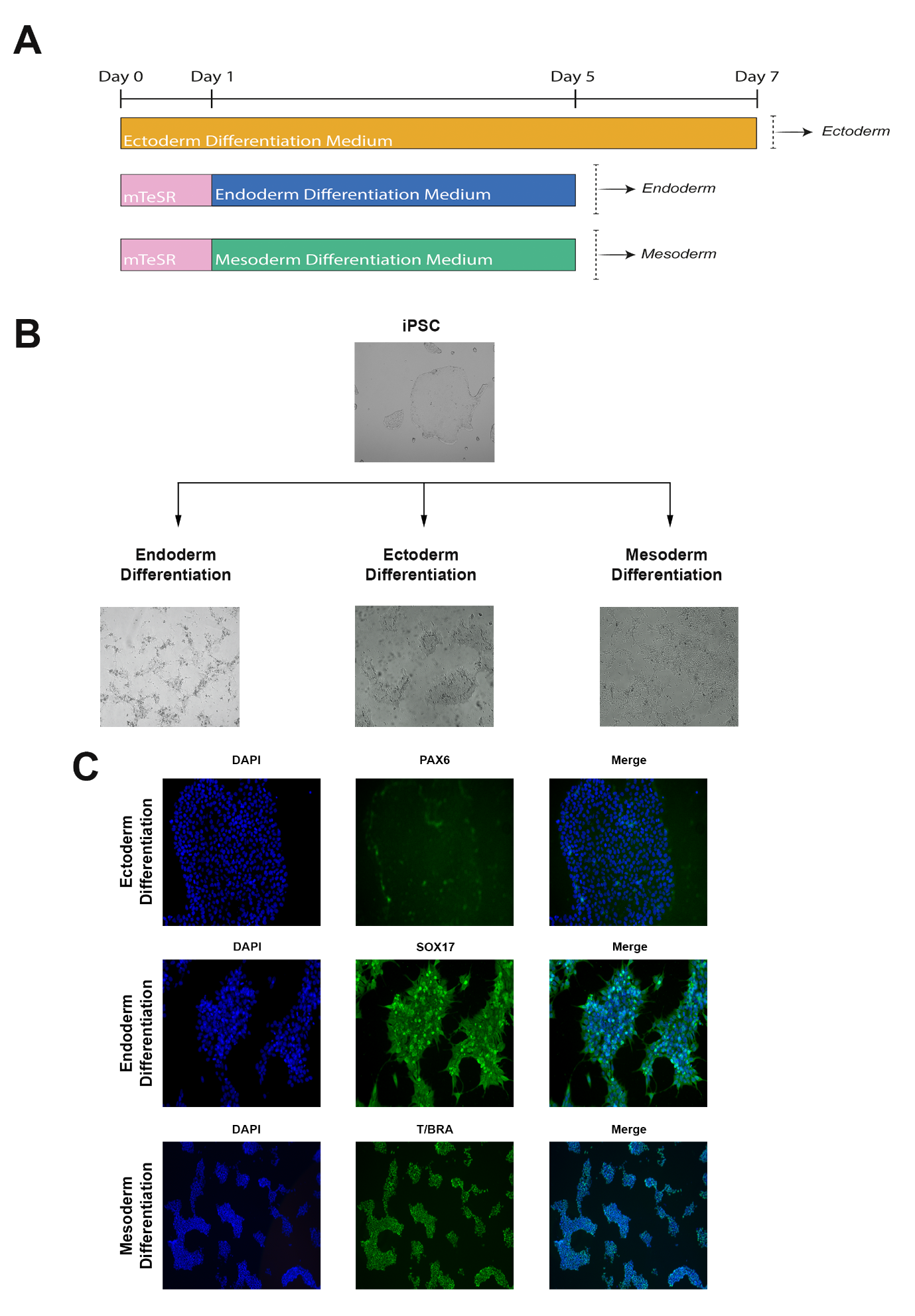


**Supplementary Figure 1.** Assessment of the tri-lineage differentiation potential of iPSCs.

A) A graphical representation of the trilineage differentiation protocol. B) Light microscopy images that are representative of the differentiated iPSC colonies, showing the distinct characteristics of the ectoderm, endoderm, and mesoderm. C) Immunostaining of PAX6 (ectoderm), Sox17 (endoderm), and T/Bra (mesoderm) markers was conducted using fluorescence microscopy on cells that had undergone differentiation. n = 3


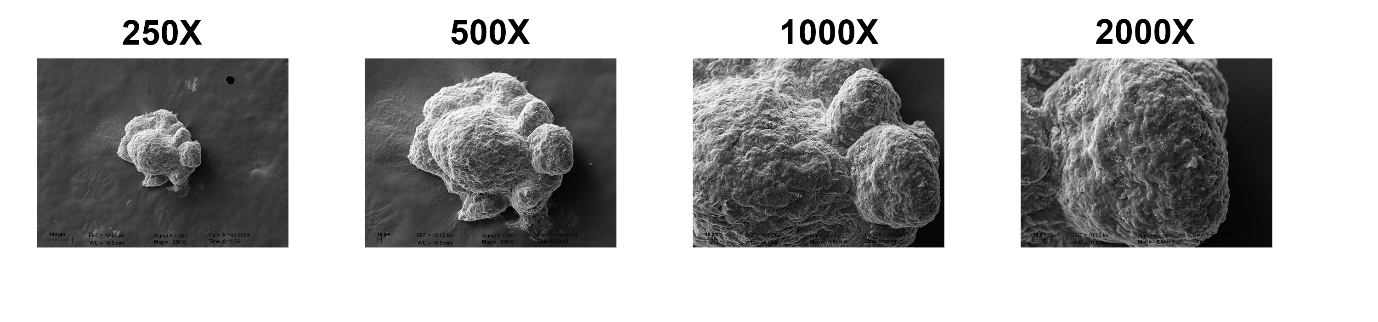


**Supplementary Figure 2.** Scanning electron microscopy images of parathyroid organoids at various magnifications. Scale bar: 100 µm, 10 µm.


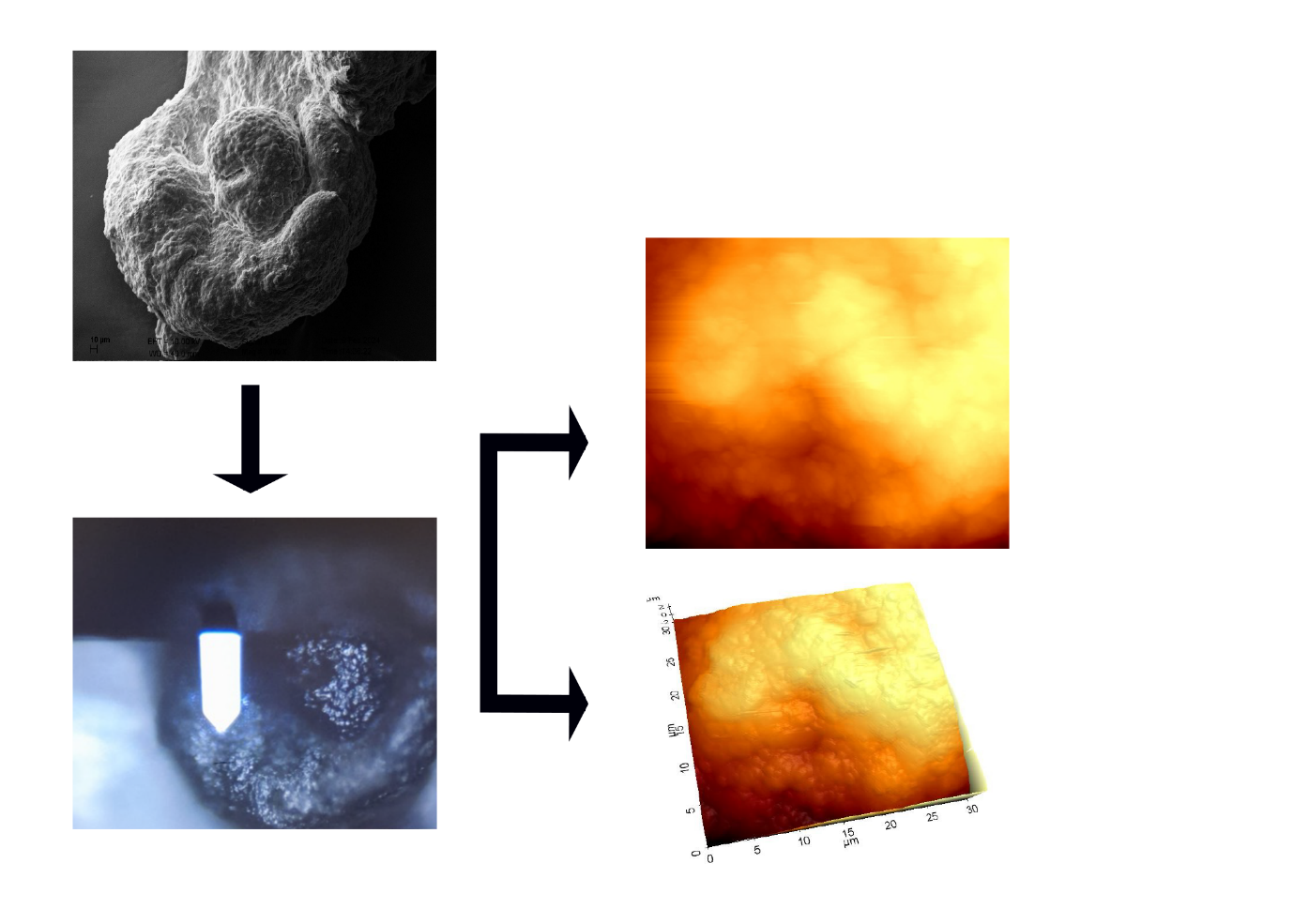


**Supplementary Figure 3.** Atomic force microscopy analysis of parathyroid organoids. AFM analysis was performed at the indicated area detected by scanning electron microscope.


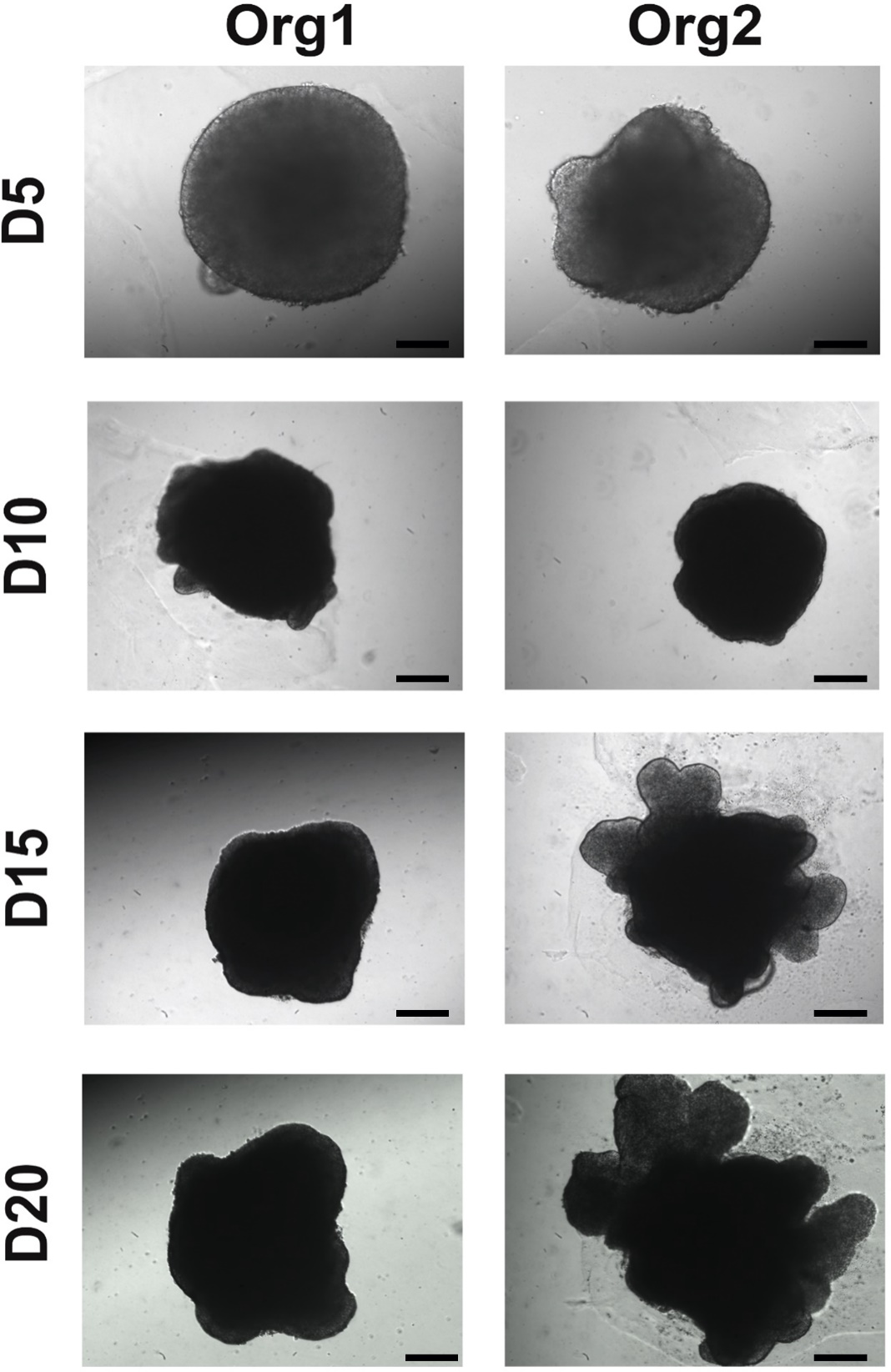


**Supplementary Figure 4.** Assessment of organoid formation potential by two different generation techniques for 20 days. Org1 represents one single organoid embedded in one Matrigel dome. Org2 represents one organoid of a Matrigel dome in which multiple organoids were embedded. n = 3, Scale bar: 100 µm.


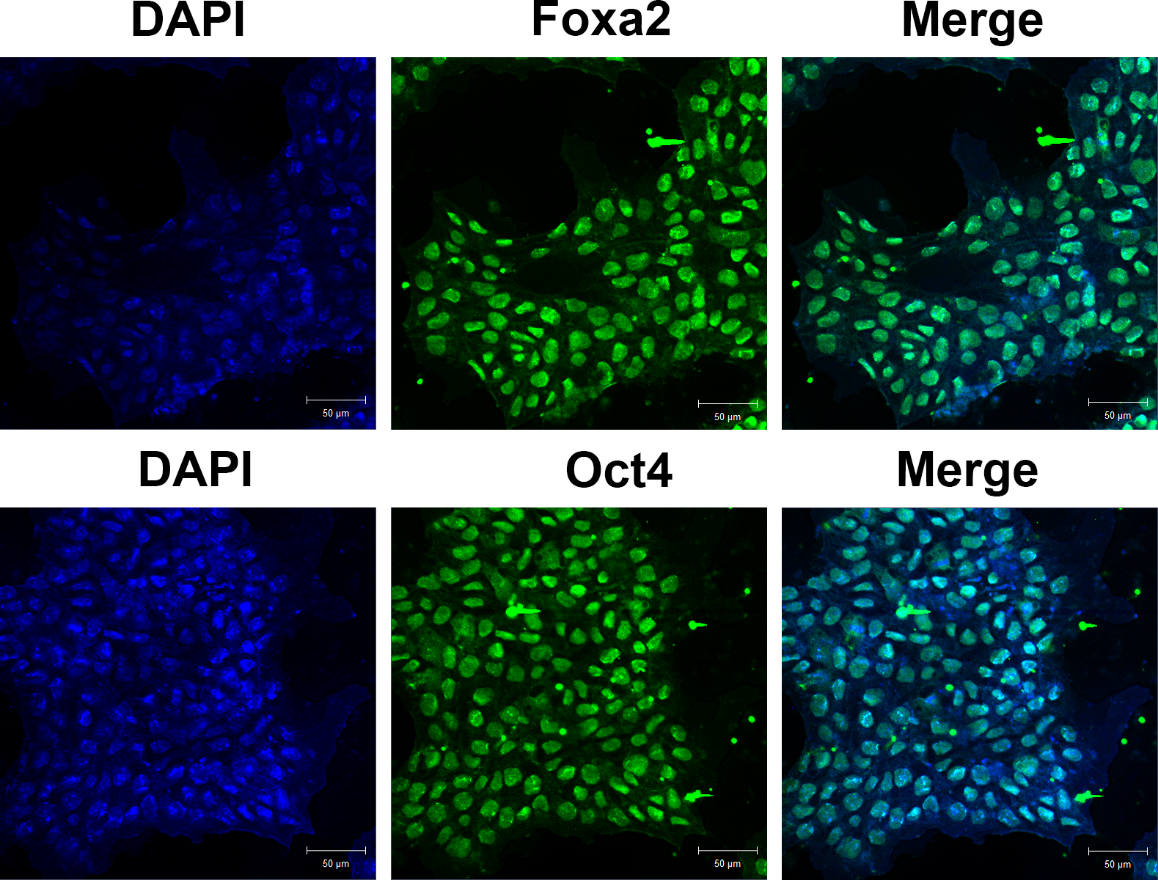


**Supplementary Figure 5.** Staining of Foxa2 and Oct4 expression in cells at Day 0.

n = 3, Scale bar: 50 µm


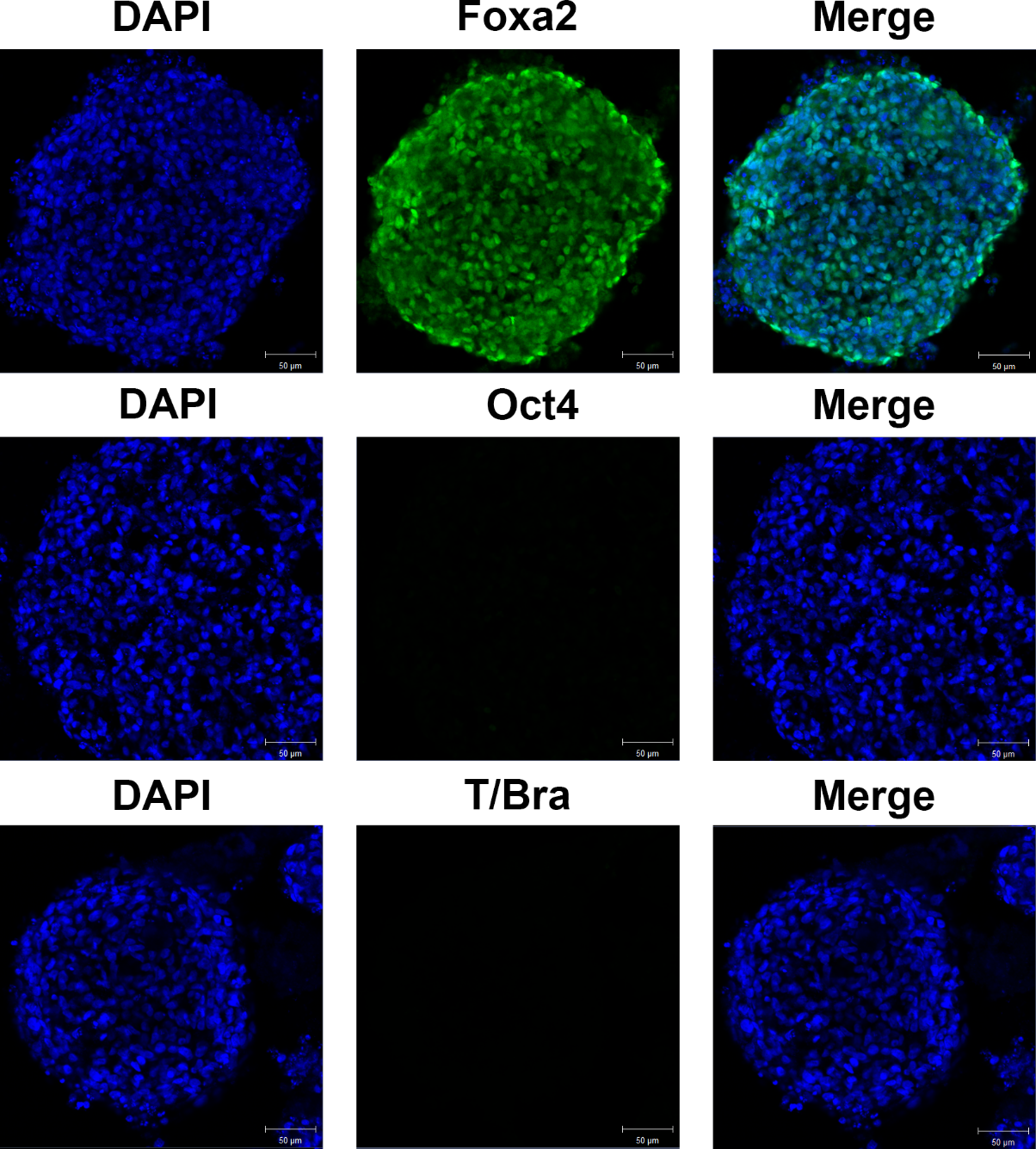


**Supplementary Figure 6.** Staining of Foxa2, Oct4, and T/Bra expression in cells at

Day 3. n = 3, Scale bar: 50 µm


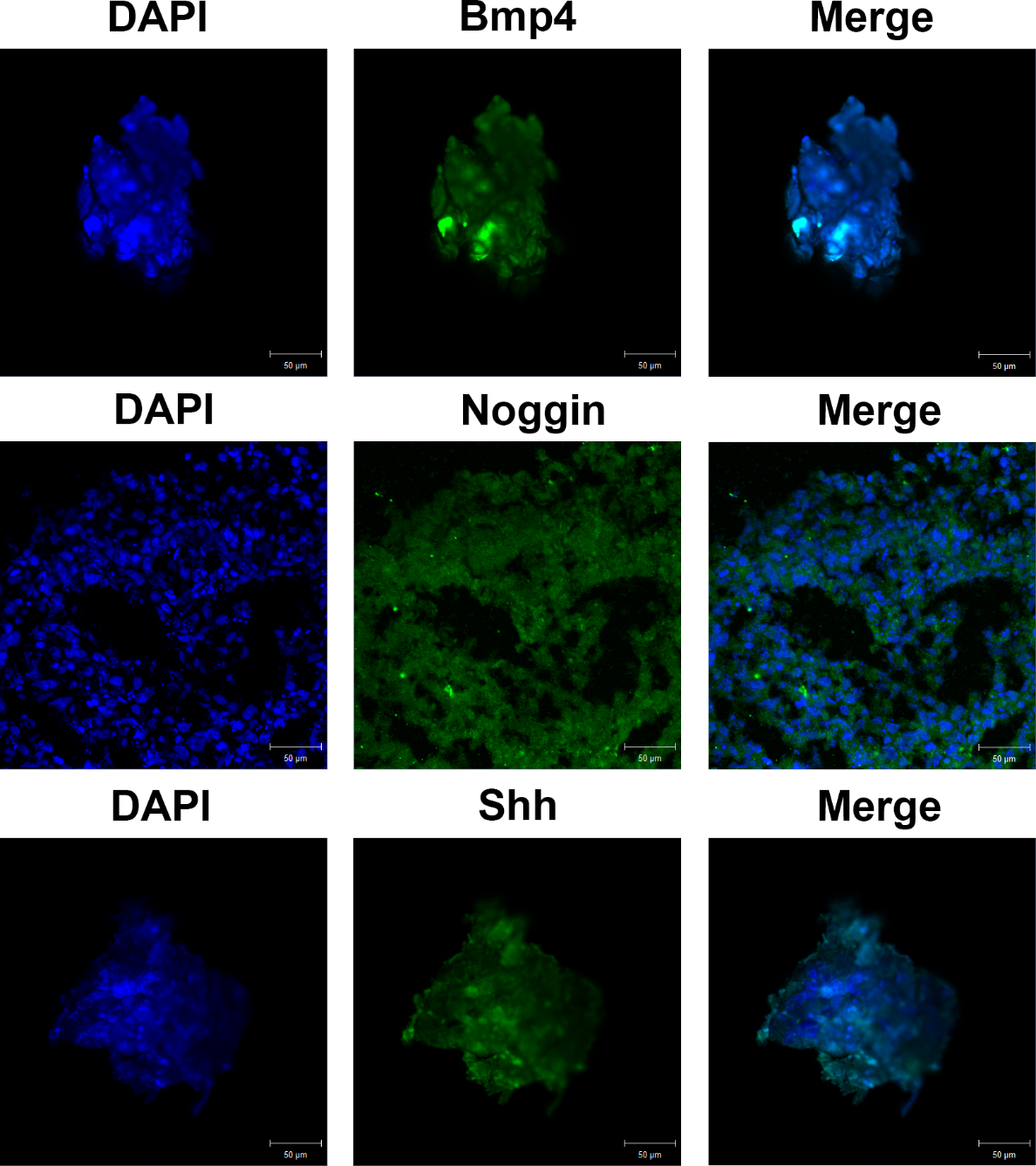


**Supplementary Figure 7.** Staining of Bmp4, Noggin and Shh expression in organoids at Day 5. n = 3, Scale bar: 50 µm


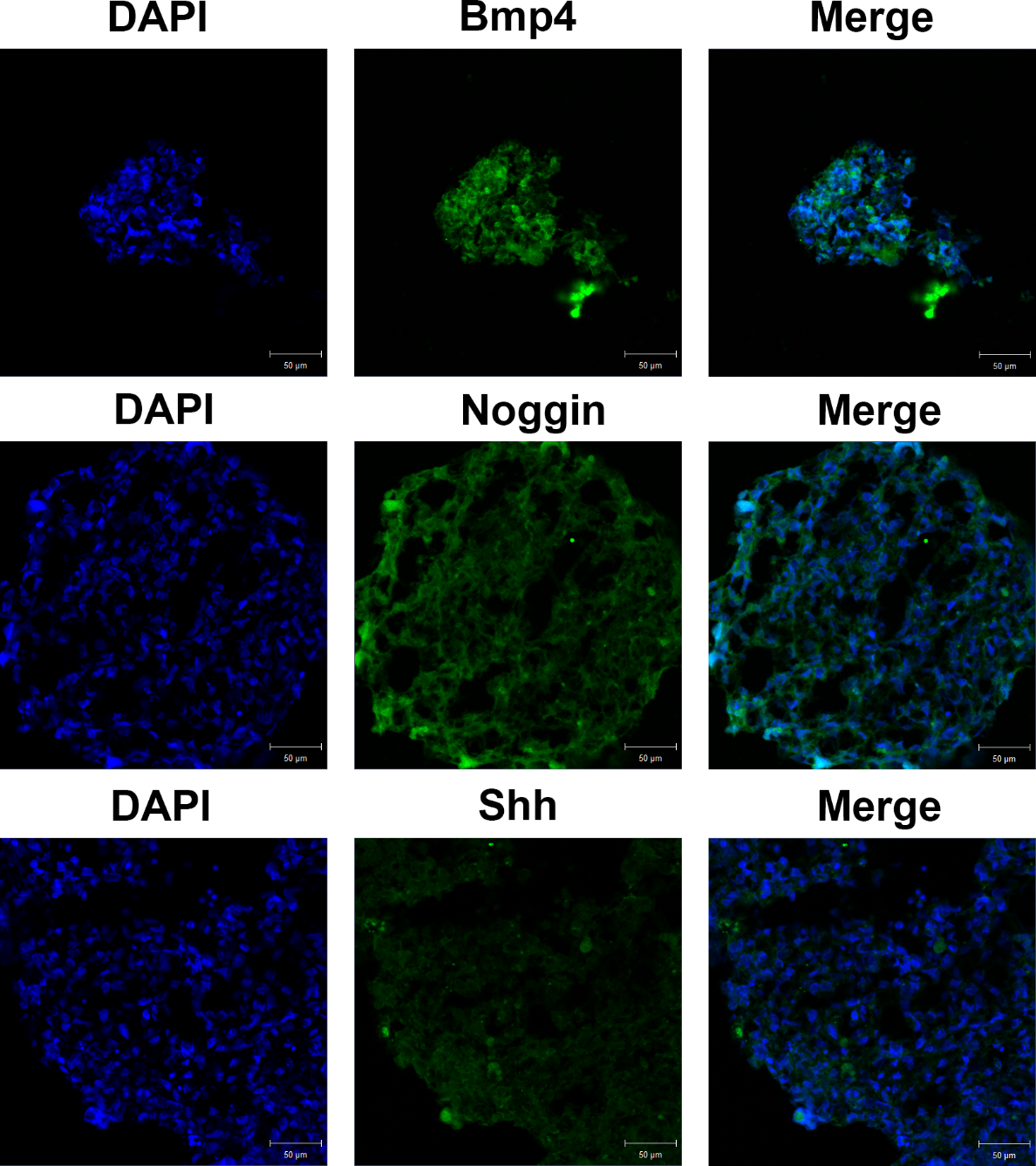


**Supplementary Figure 8.** Staining of Bmp4, Noggin and Shh expression in organoids at Day 10. n = 3, Scale bar: 50 µm


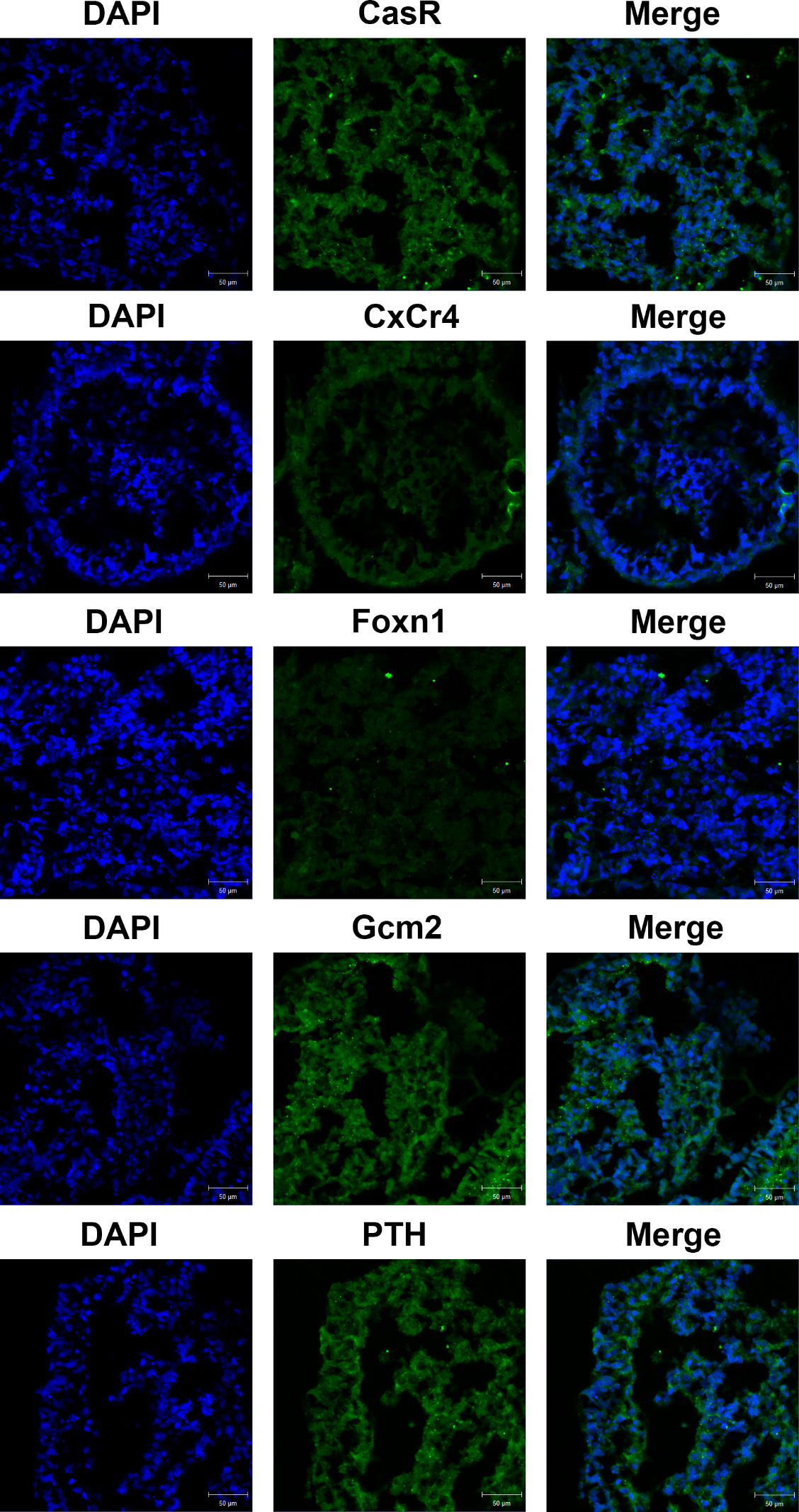


**Supplementary Figure 9.** Staining of CasR, CxCr4, Foxn1, Gcm2, and PTH expression in organoids at Day 15. n = 3, Scale bar: 50 µm


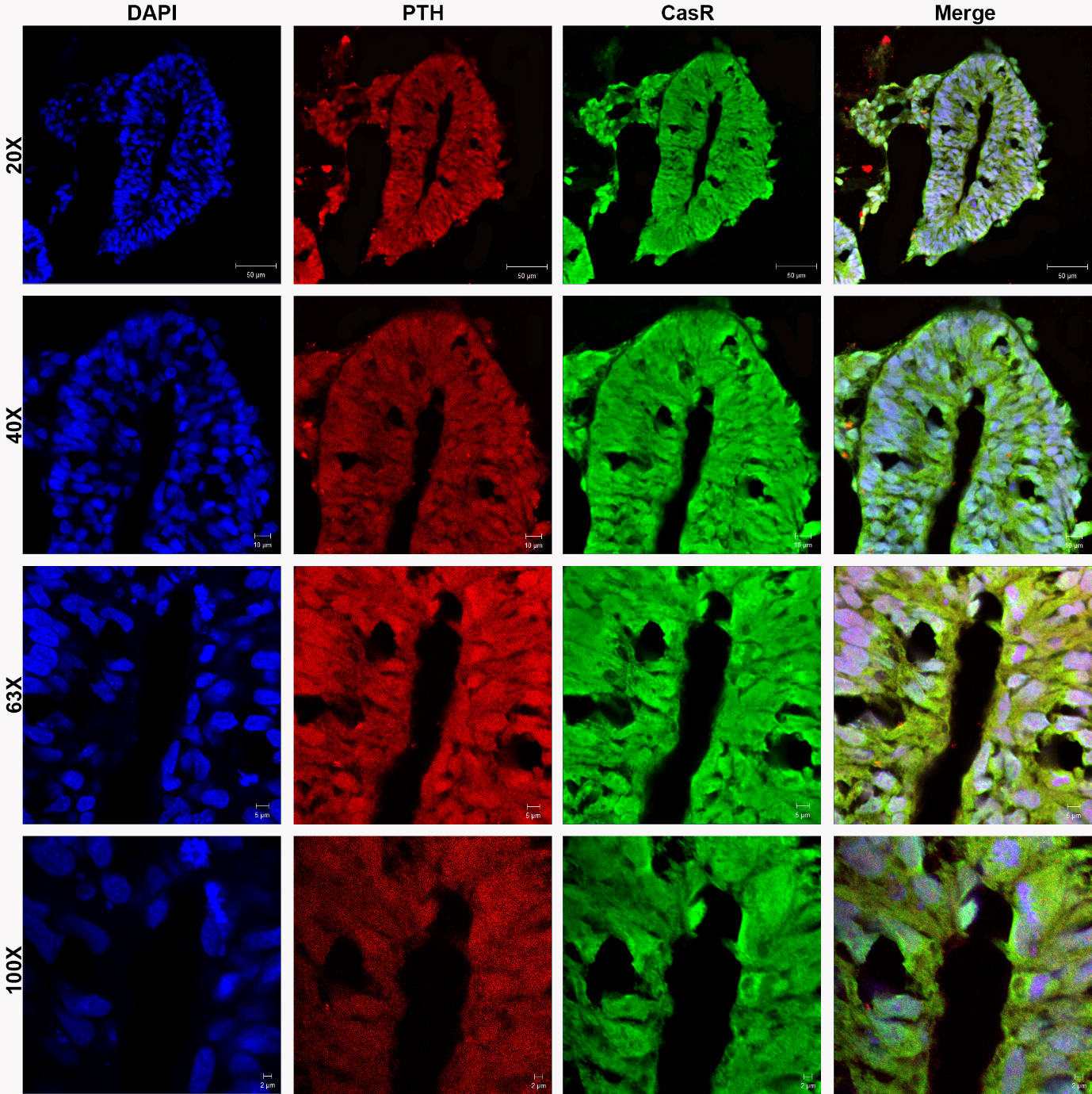


**Supplementary Figure 10.** Double staining of PTH and CasR expression in organoids at Day 20. n = 3, Scale bar: 50 µm, 10 µm, 5 µm, and 2 µm.


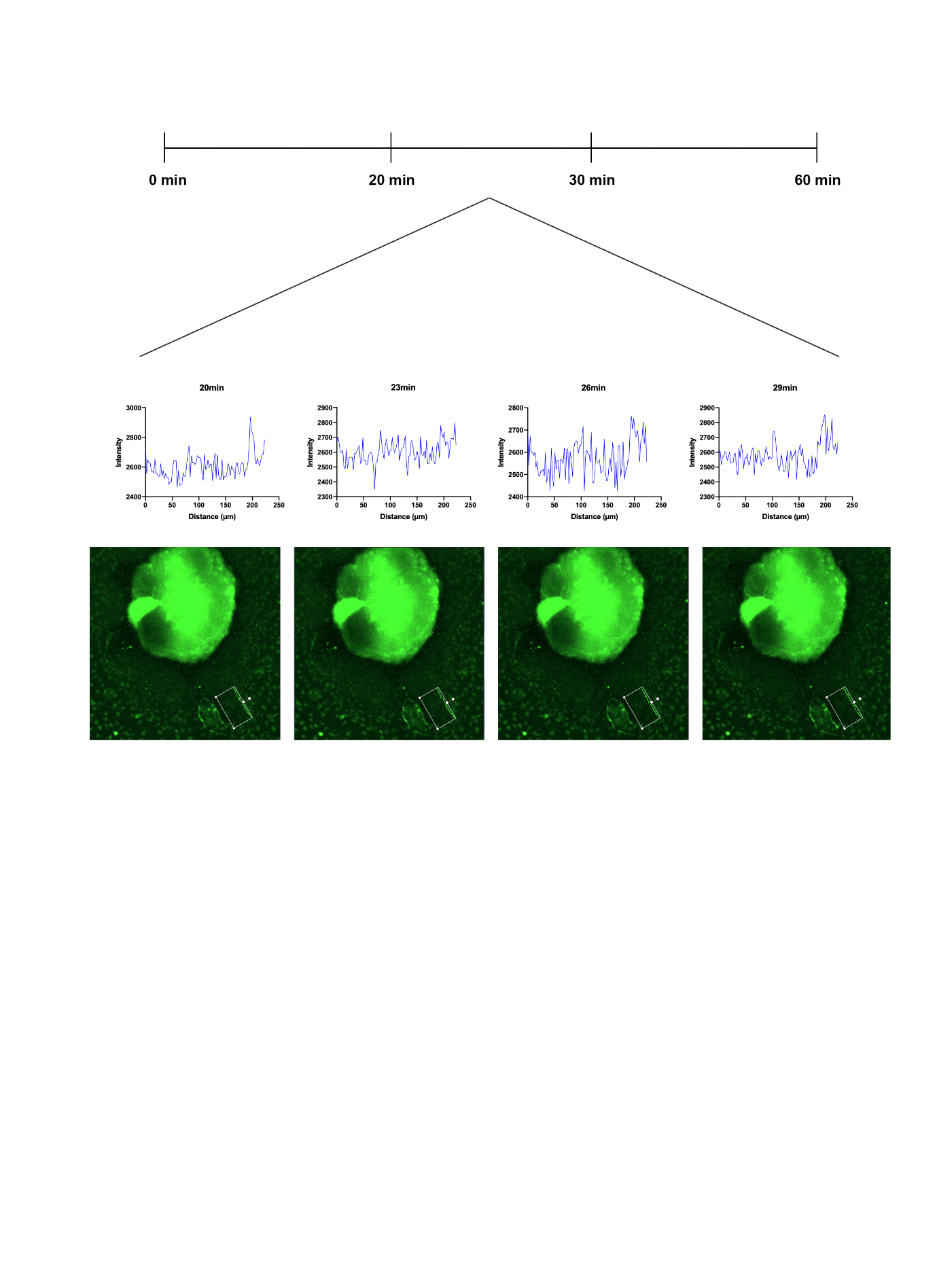


**Supplementary Figure 11.** Representative traces of Fluo-4 recordings indicate Ca^2+^ signaling in one region of parathyroid organoids. The rectangular region displayed the movement of calcium within a certain cell over a period of minutes.


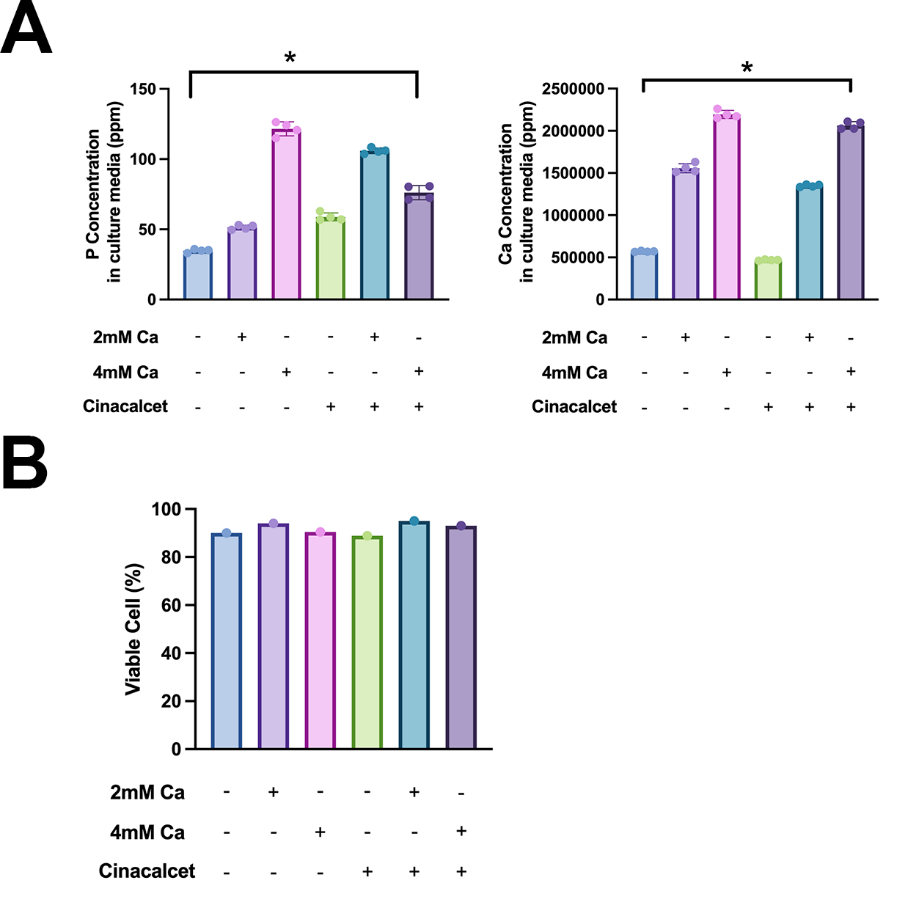


**Supplementary Figure 12.** Response of parathyroid organoids to calcium and cinacalcet. A) Phosphorous and calcium concentration in culture media of day 20 parathyroid organoid in the presence of 2 mM, and 4 mM calcium and cinacalcet. Data presented as mean ± SD, n = 4, and p-values are calculated using one-way ANOVA, *p<0.05. B) Viable cell numbers of day 20 parathyroid organoid in the presence of 2 mM, and 4 mM calcium and cinacalcet, n = 2.


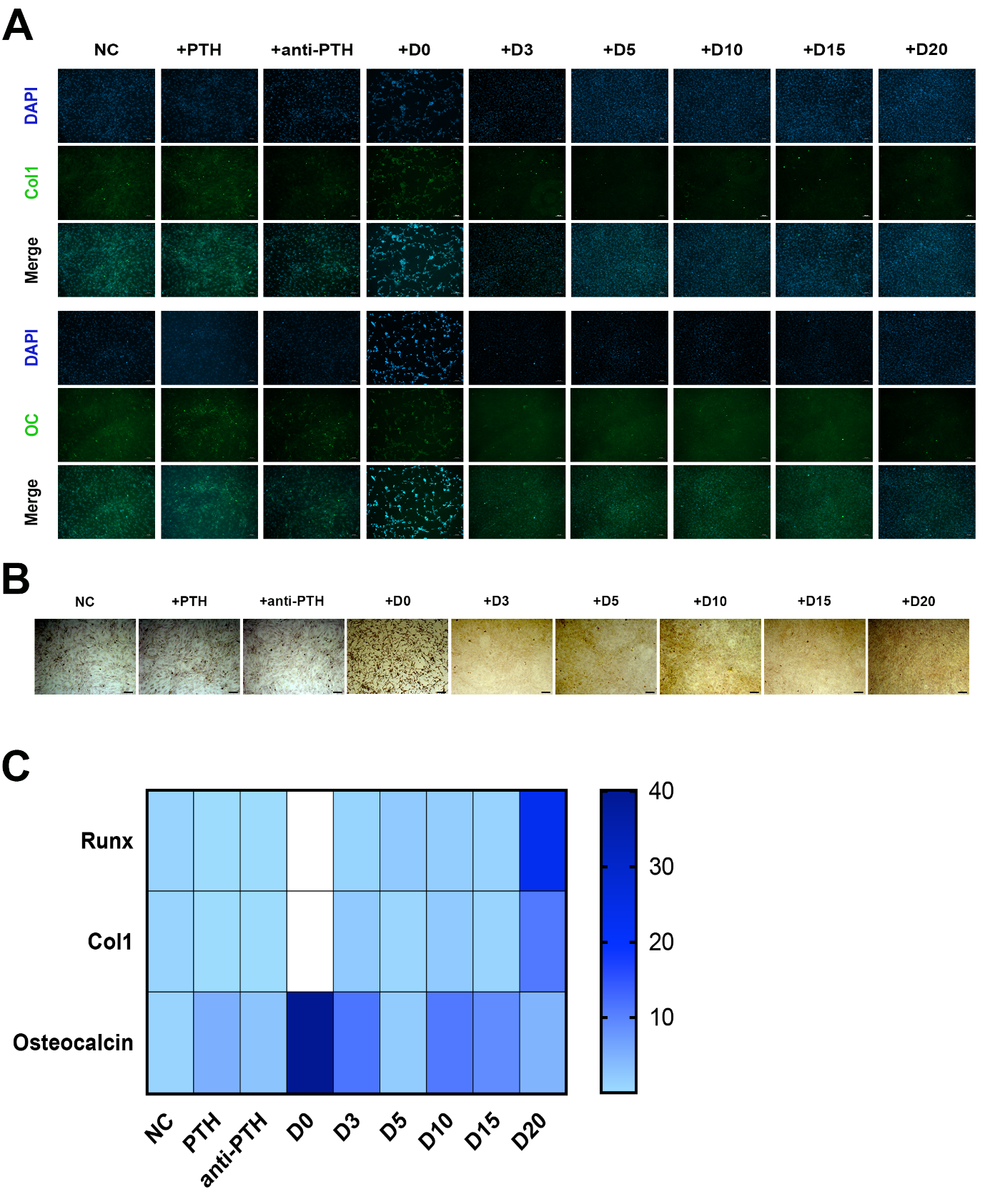


**Supplementary Figure 13.** The effect of organoid secretome on osteogenic differentiation in vitro. A) The immunofluorescence staining images reveal the cellular translocation of Col1 and OC in MC3T3-E1 pre-osteoblast cells and related experimental groups, n = 3, Scale bar: 100 µm, Col1: Collagen Type I, OC: Osteocalcin. B) Alizarin red S staining (red), with mineralized areas for each group, n = 3, Scale bar: 100 µm**.** C) qPCR showing relative mRNA levels of Runx, Col1, and osteocalcin in each group, n = 3


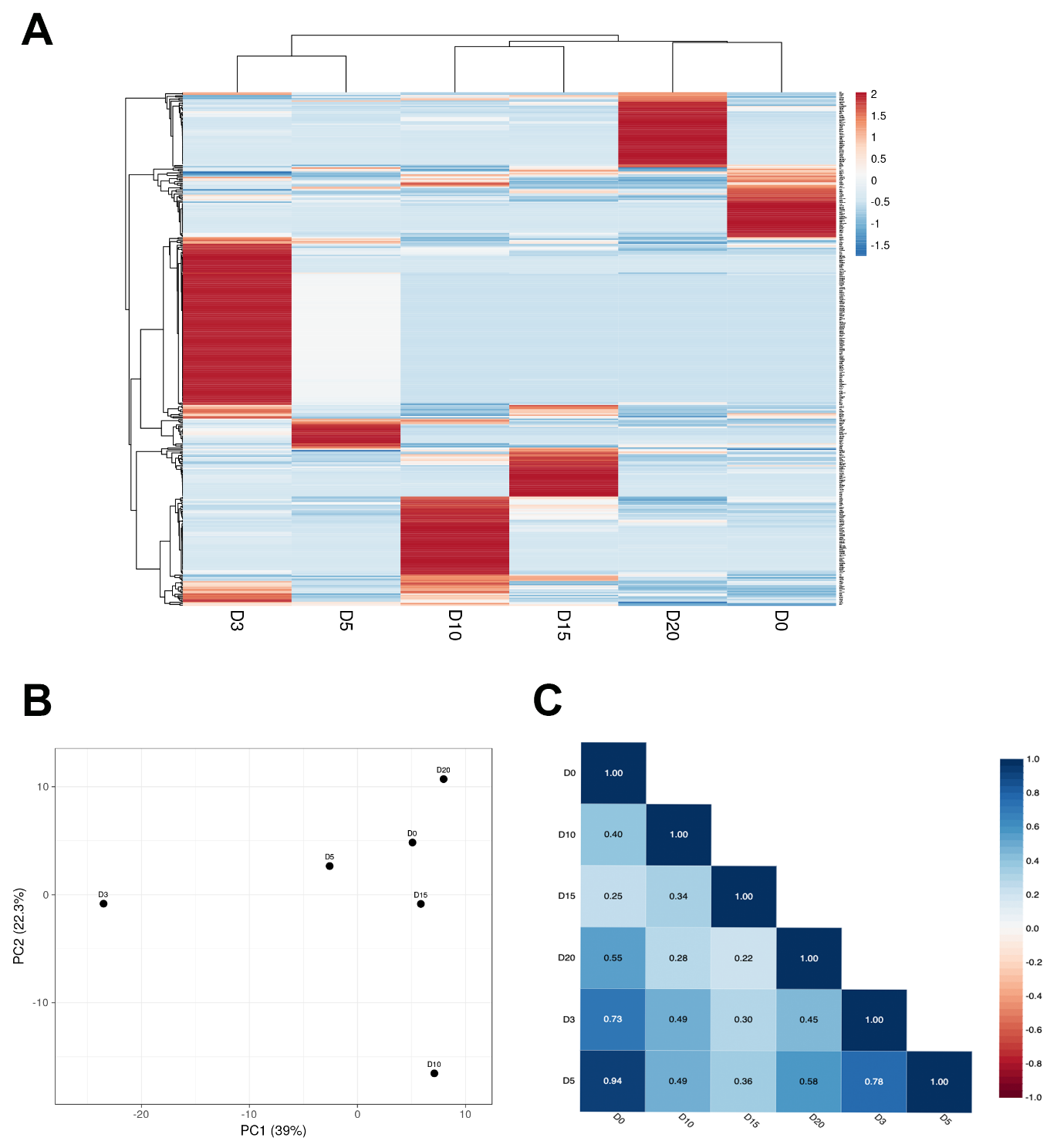


**Supplementary Figure 14.** Molecular analysis of the differentiation process in in vitro samples. A) Hierarchical clustering analysis of the genes representing the differentiation process from iPSC to parathyroid organoid, n = 2. B) PCA analysis of RNA-seq for day-by-day samples across two replicates, based on the 500 most variable genes, n = 2. x-axis: PC1; y-axis: PC2. C) Correlation matrix of Pearson correlation analysis of RNA-Seq results, n = 2.


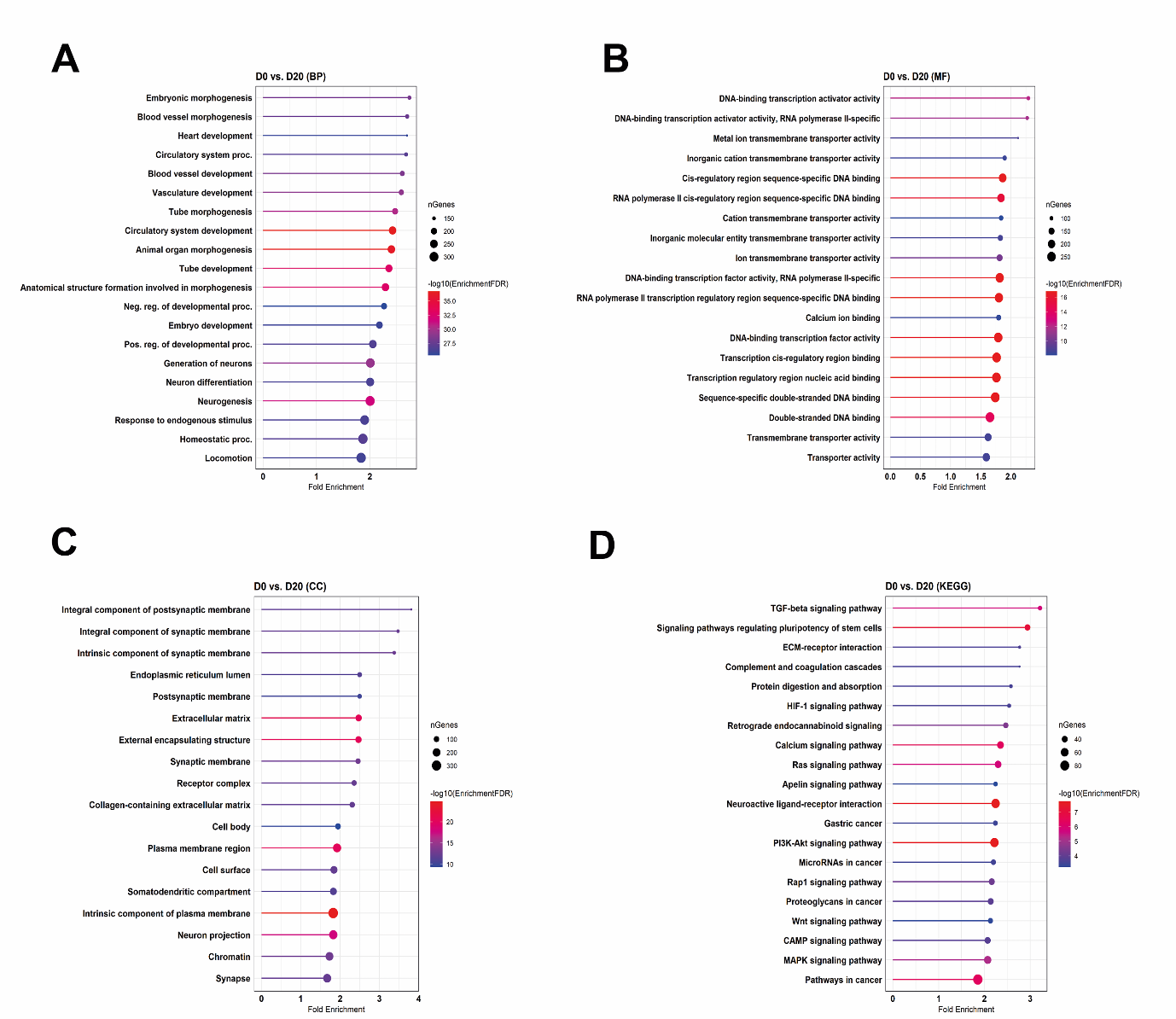


**Supplementary Figure 15.** Gene ontology (GO) analysis of RNA-Seq between D0 (iPSC) and D20 (Parathyroid organoid) for A) Biological process, B) Molecular function, C) Cellular component, and D) KEGG pathway, n = 2.


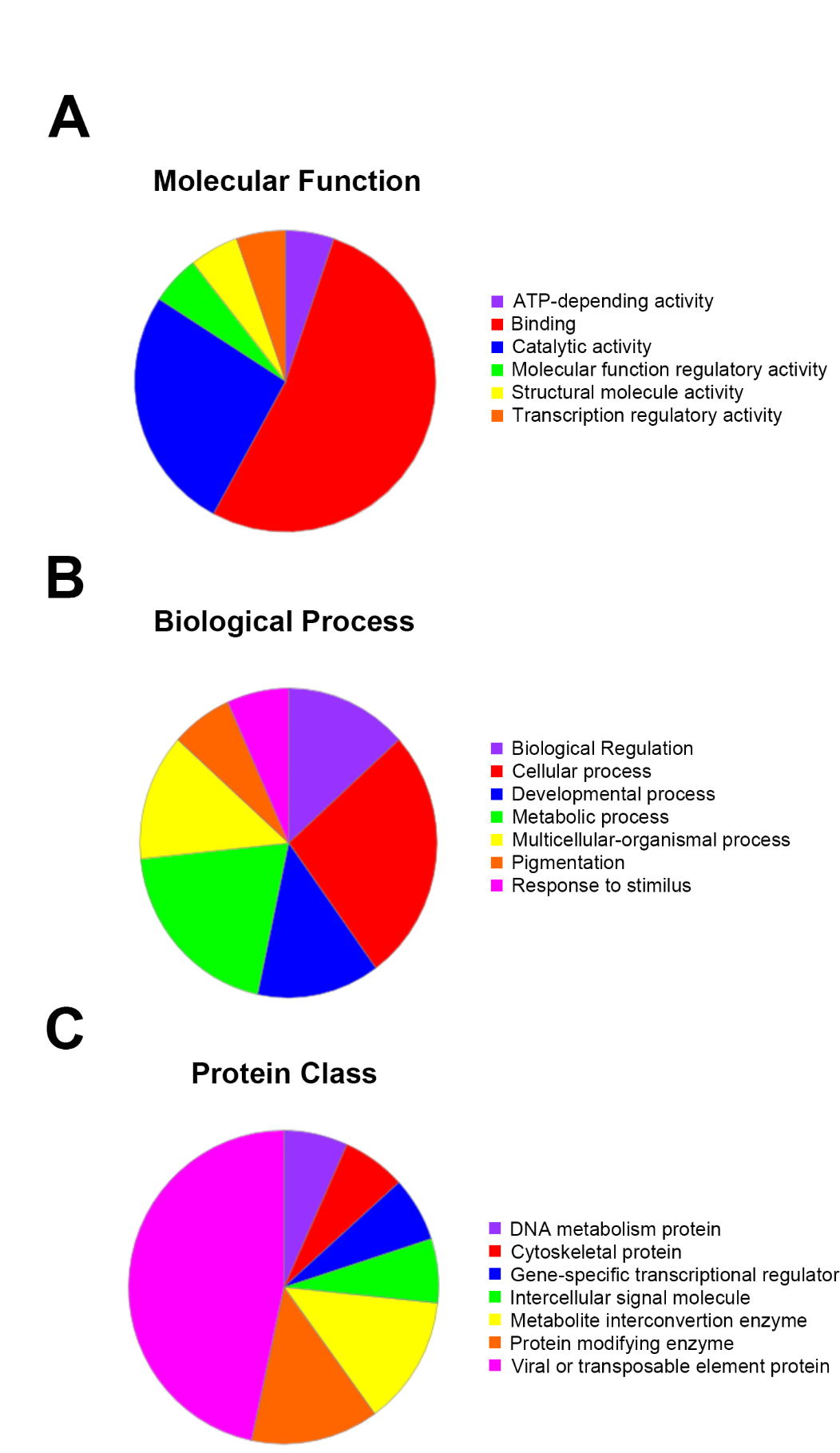


**Supplementary Figure 16.** Analysis of GO using PANTHER for A) Molecular function, B) Biological process, and C) Protein class, n = 2.


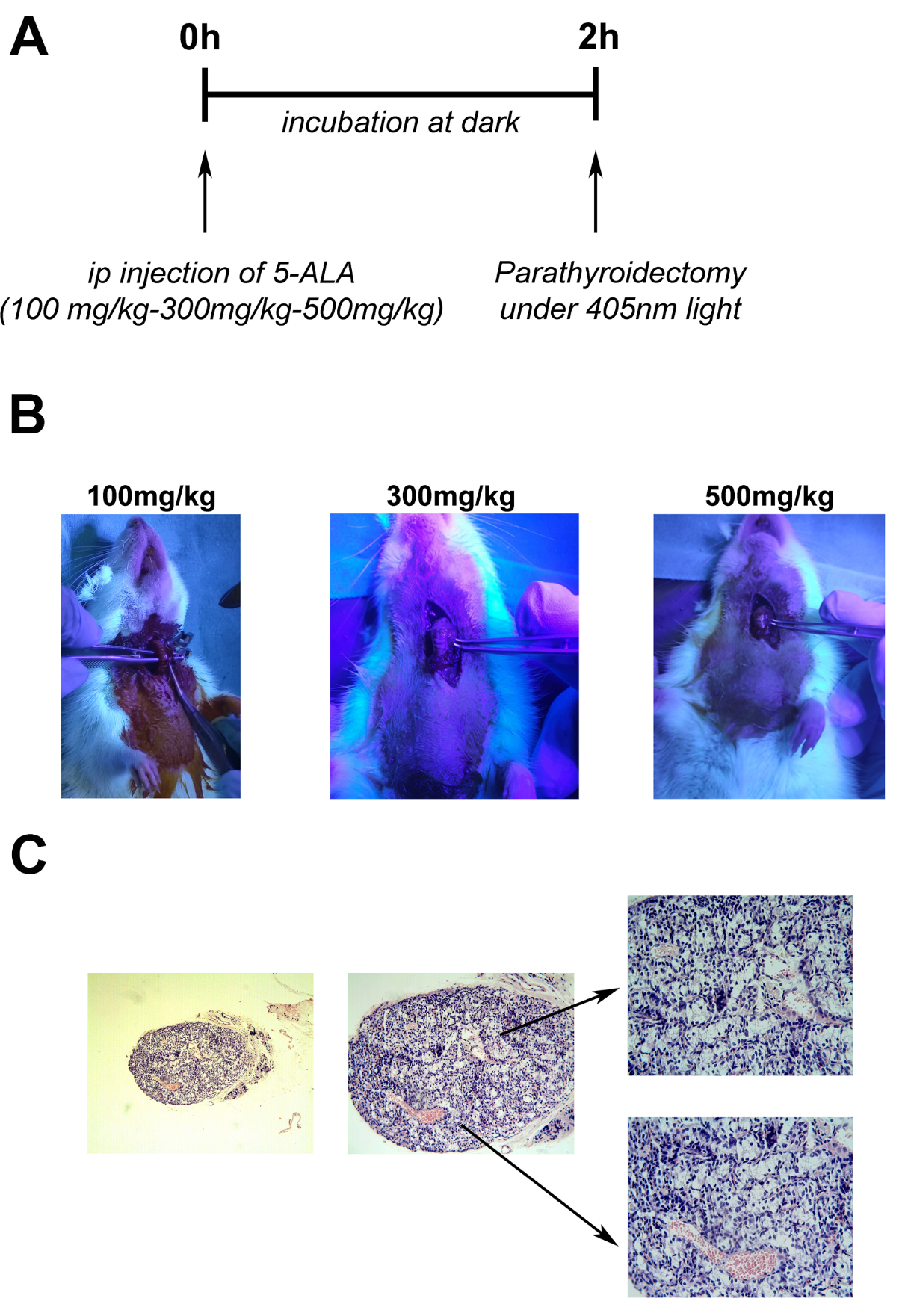


**Supplementary Figure 17.** Optimization of 5-aminolevulinic acid (5-ALA) concentration and parathyroidectomy model generation. A) 5-ALA injection and visualization timeline. B) The images were captured under 405 nm light by a regular photograph camera after the intraperitoneal injection of 5-ALA and 2 h incubation at dark. C) Image of extracted parathyroid tissue stained with hematoxylin and eosin following parathyroidectomy.


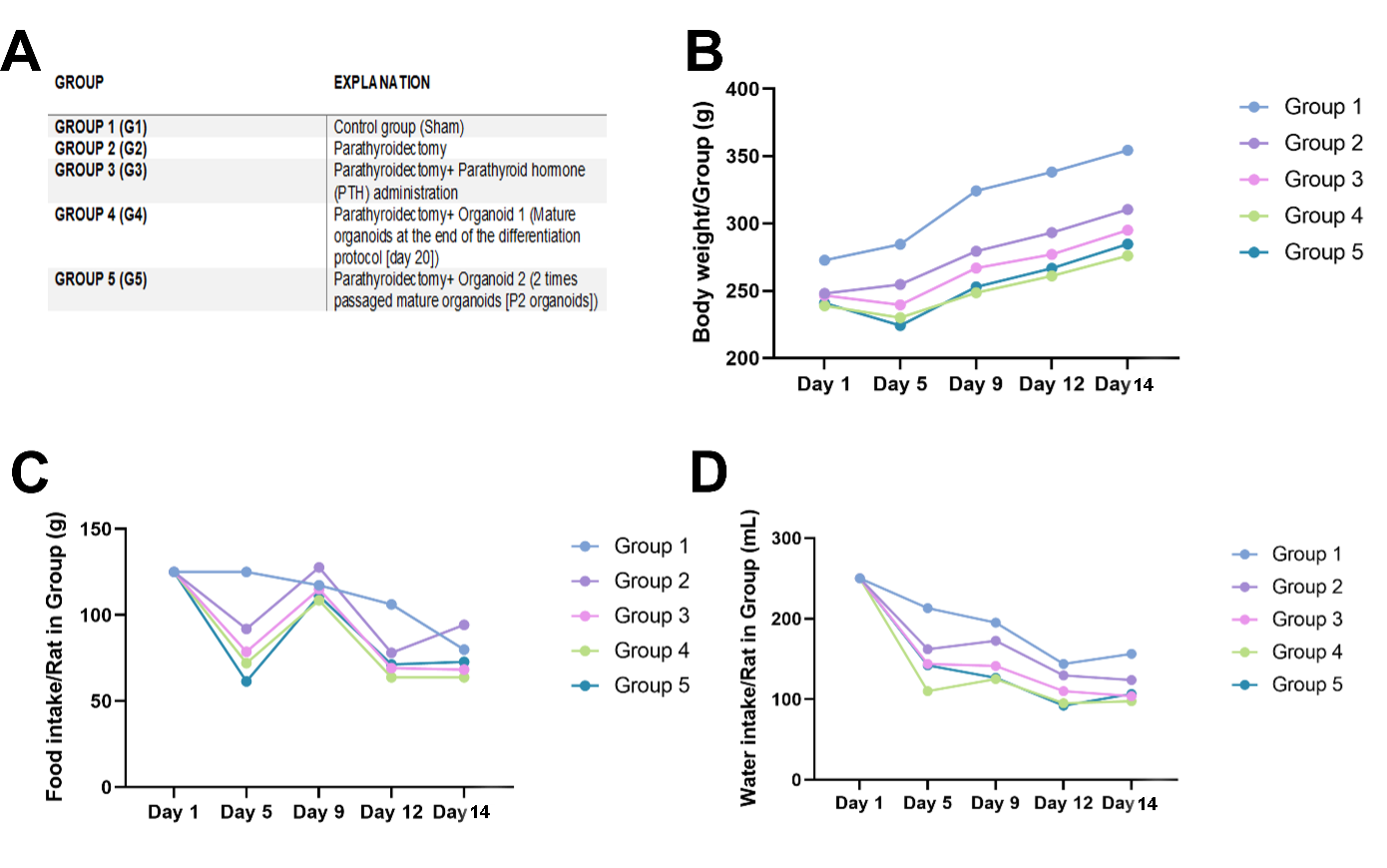


**Supplementary Figure 18**. Analyses and experimental groups in vivo after parathyroid organoid transplantation. A) Animal experiments group descriptions (n = 8). B) Body weight; C) Dietary intake; D) Water consumption of animals during animal experiments.


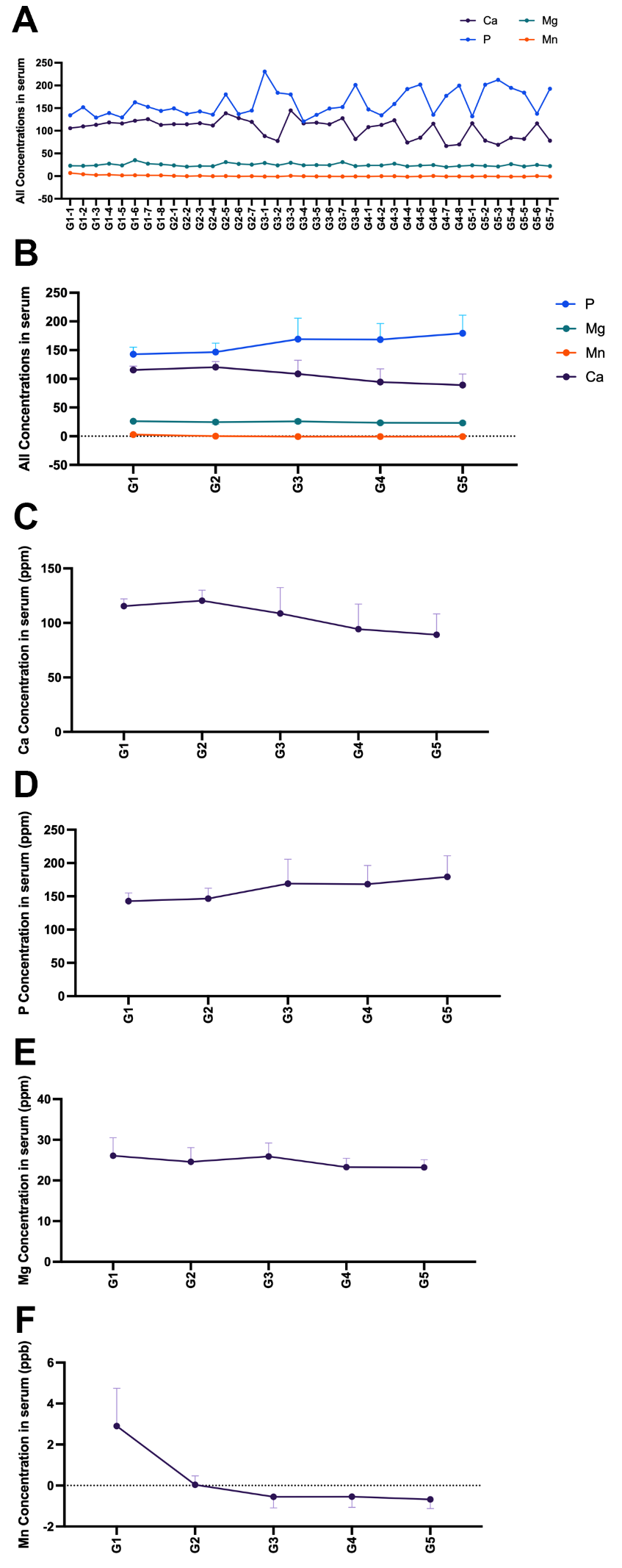


**Supplementary Figure 19.** ICP-MS functional analyses in vivo after parathyroid organoid transplantation. A) Concentrations of calcium, phosphate, magnesium, and manganese in the serum of every rat involved in the experiment. B) Average concentrations of calcium, phosphate, magnesium, and manganese in the serum of animals. C) Average concentrations of calcium in the serum of each group. D) Average concentrations of phosphate in the serum of each group. E) Average concentrations of magnesium in the serum of each group. F) Average concentrations of manganese in the serum of each group. Data presented as mean ± SD, n = 8.


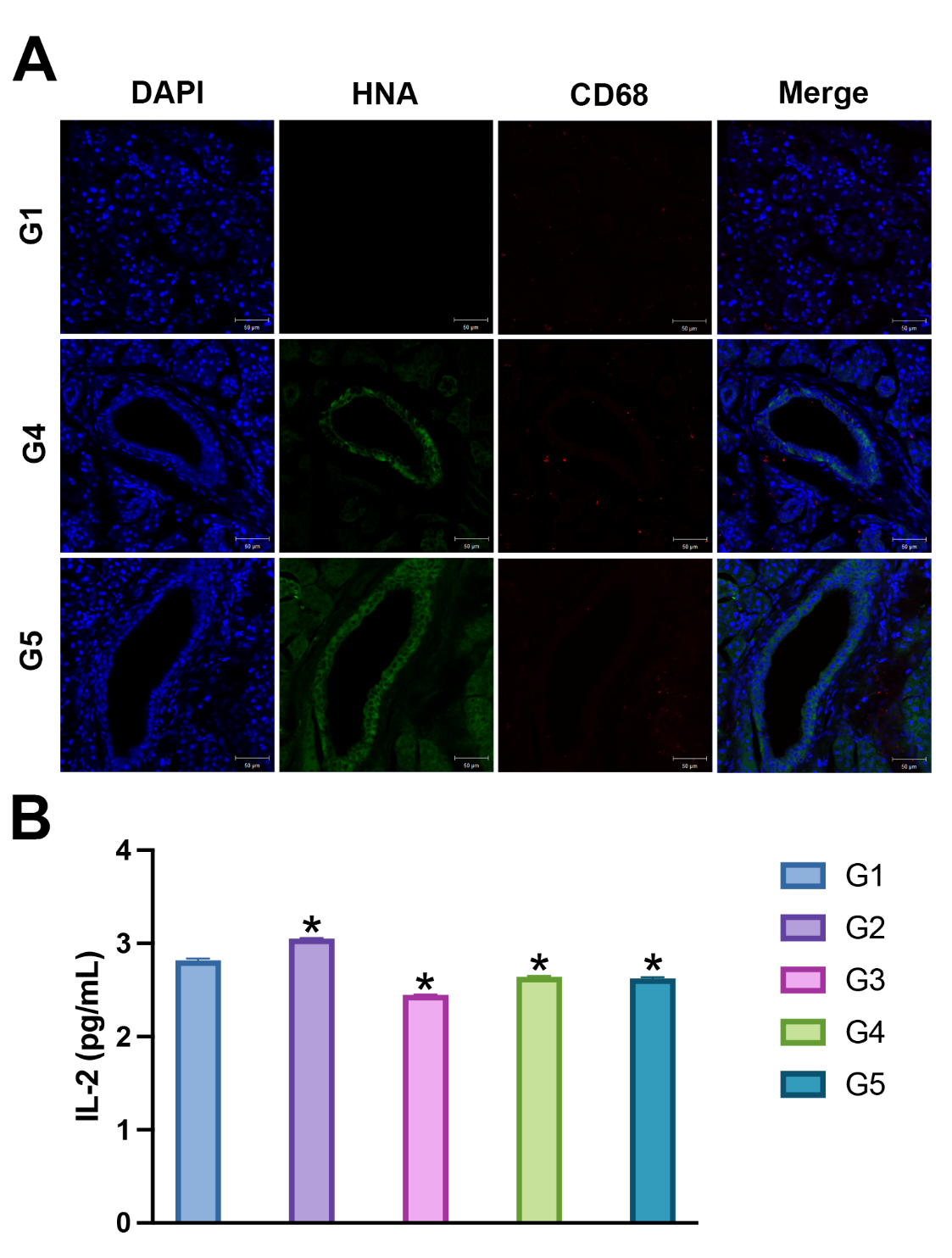


**Supplementary Figure 20.** Determination of immune response after transplantation of parathyroid organoids. A) Double staining of CD68 and HNA expressing organoids at in vivo experimental groups. HNA: Human Nuclear Antigen, n = 3, Scale bar: 50 µm. B) IL-2 (pg/mL) measurement in each rat serum. Data are presented as mean ± SD, n = 2, and p-values are calculated using one-way ANOVA, *p<0.05.


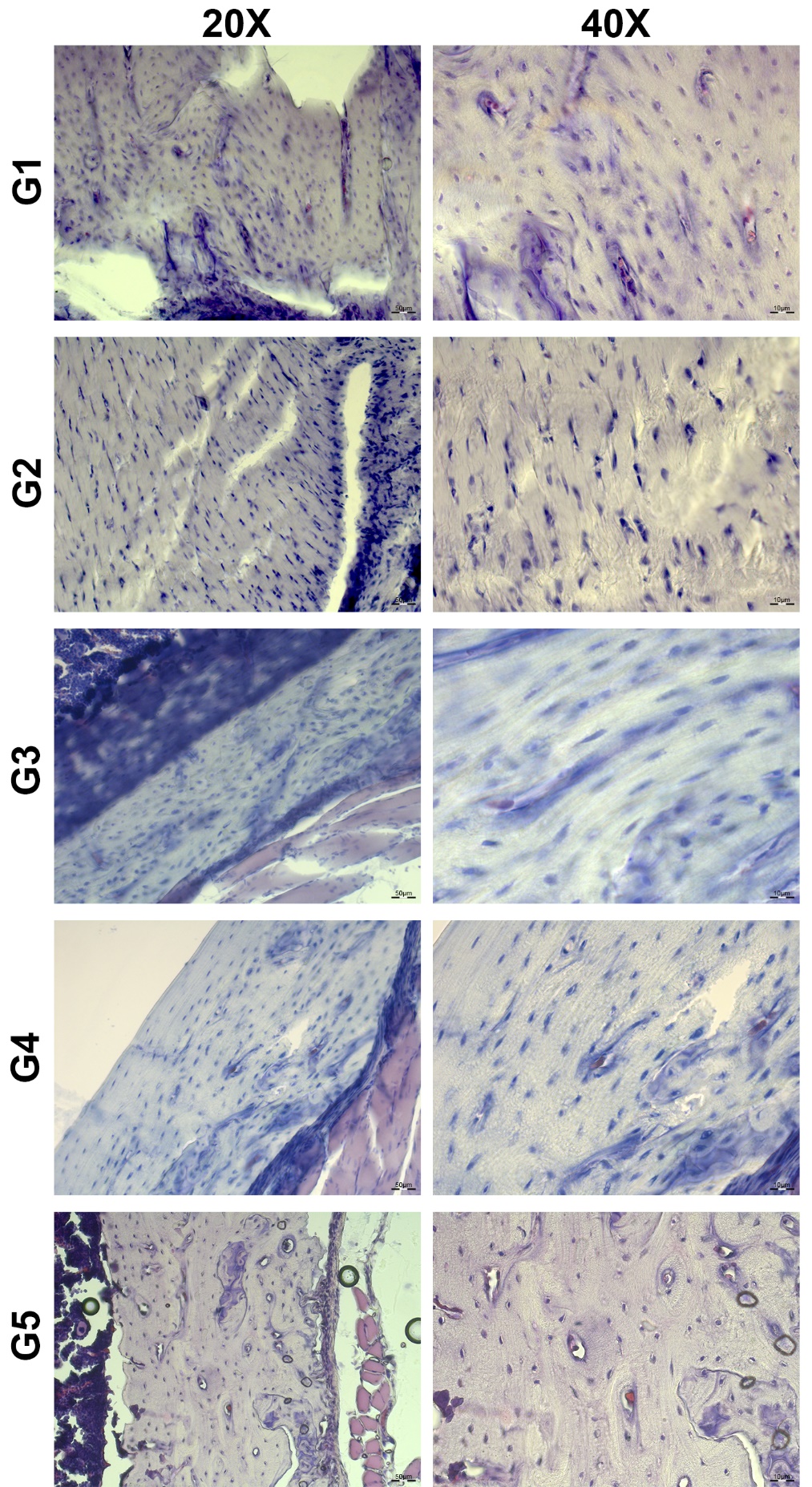


**Supplementary Figure 21.** Histological examination of rat bone slices with a thickness of 10 µm, stained with H&E, n = 3, scale bar: 50 µm, 10 µm.


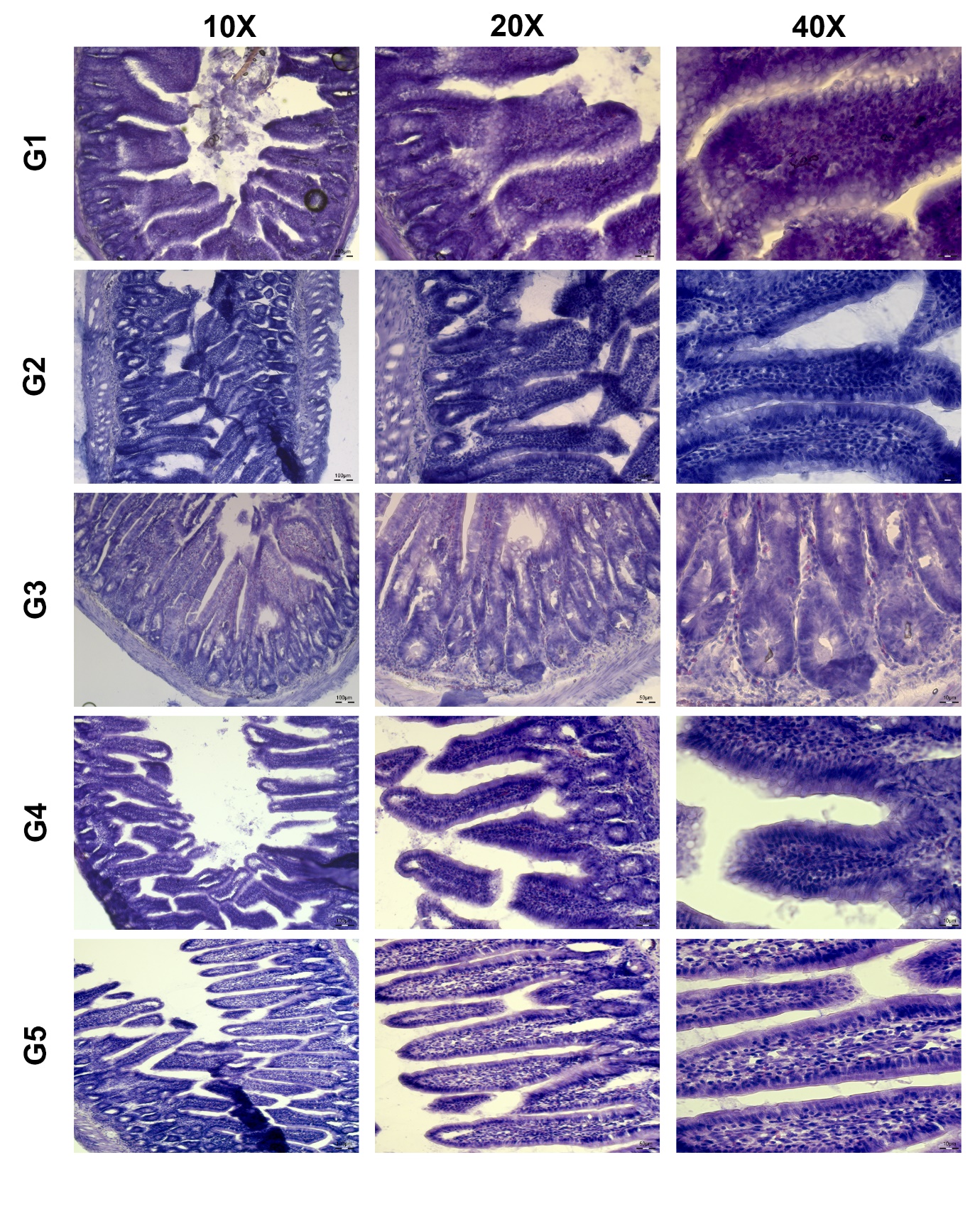


**Supplementary Figure 22.** Histological examination of rat small intestine slices with a thickness of 10 µm, stained with H&E, n = 3, scale bar: 100 µm, 50 µm, 10 µm.


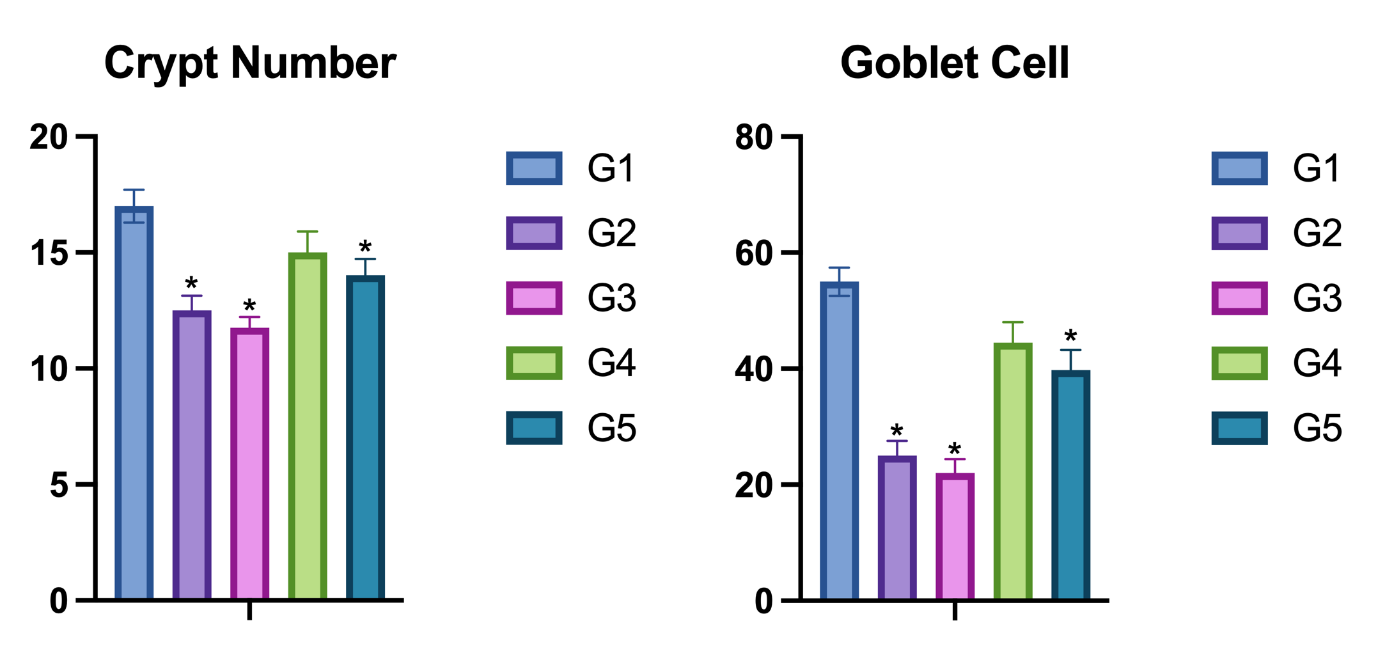


**Supplementary Figure 23.** The quantification of crypts and goblet cells in small intestinal tissues after the H&E staining. Data presented as mean ± SEM, n = 4, and p-values are calculated using one-way ANOVA, *p<0.05.


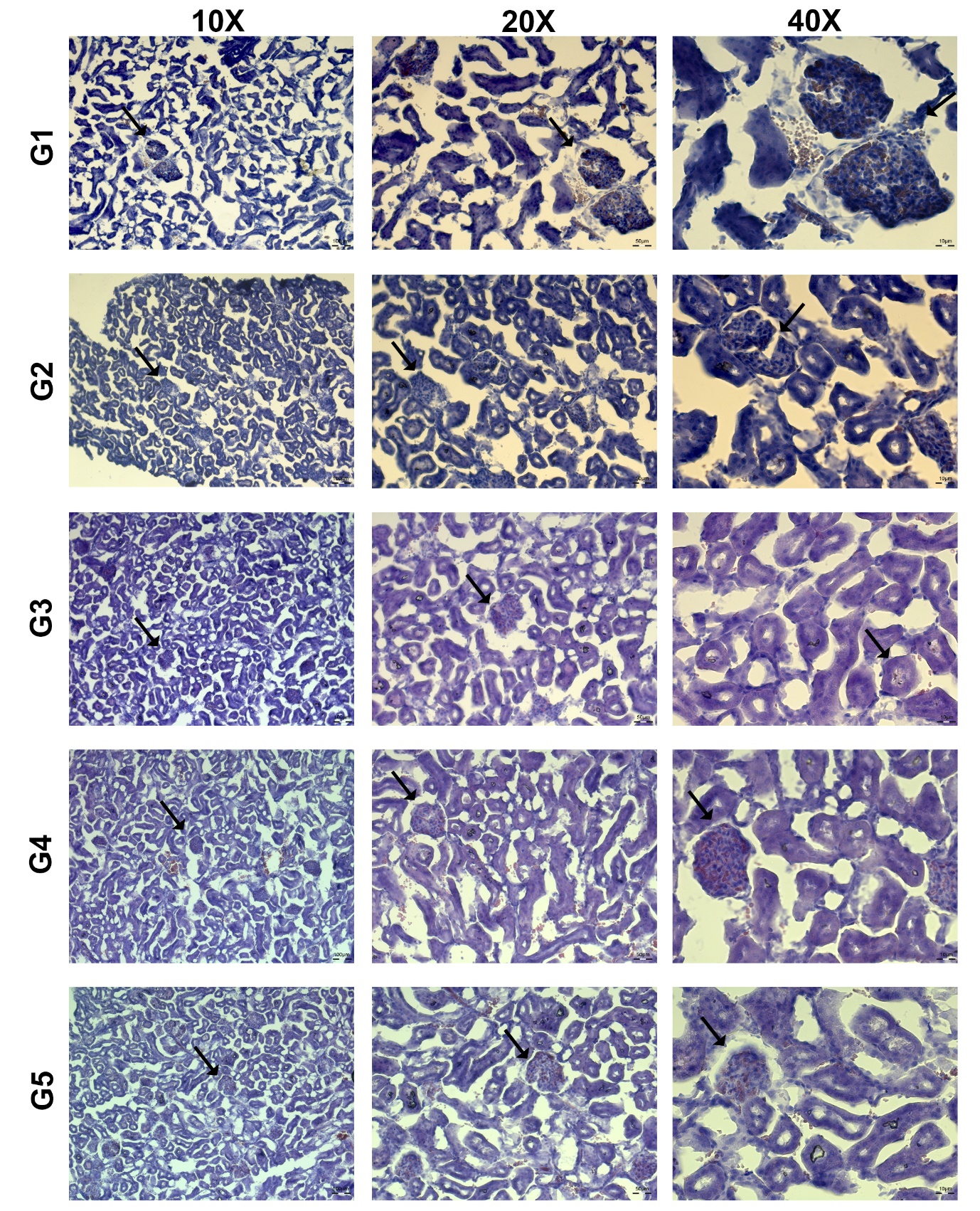


**Supplementary Figure 24.** Histological examination of rat kidney slices with a thickness of 10 µm, stained with H&E. The Bowman capsule was indicated by black arrows, n = 3, scale bar: 100 µm, 50 µm, 10 µm.


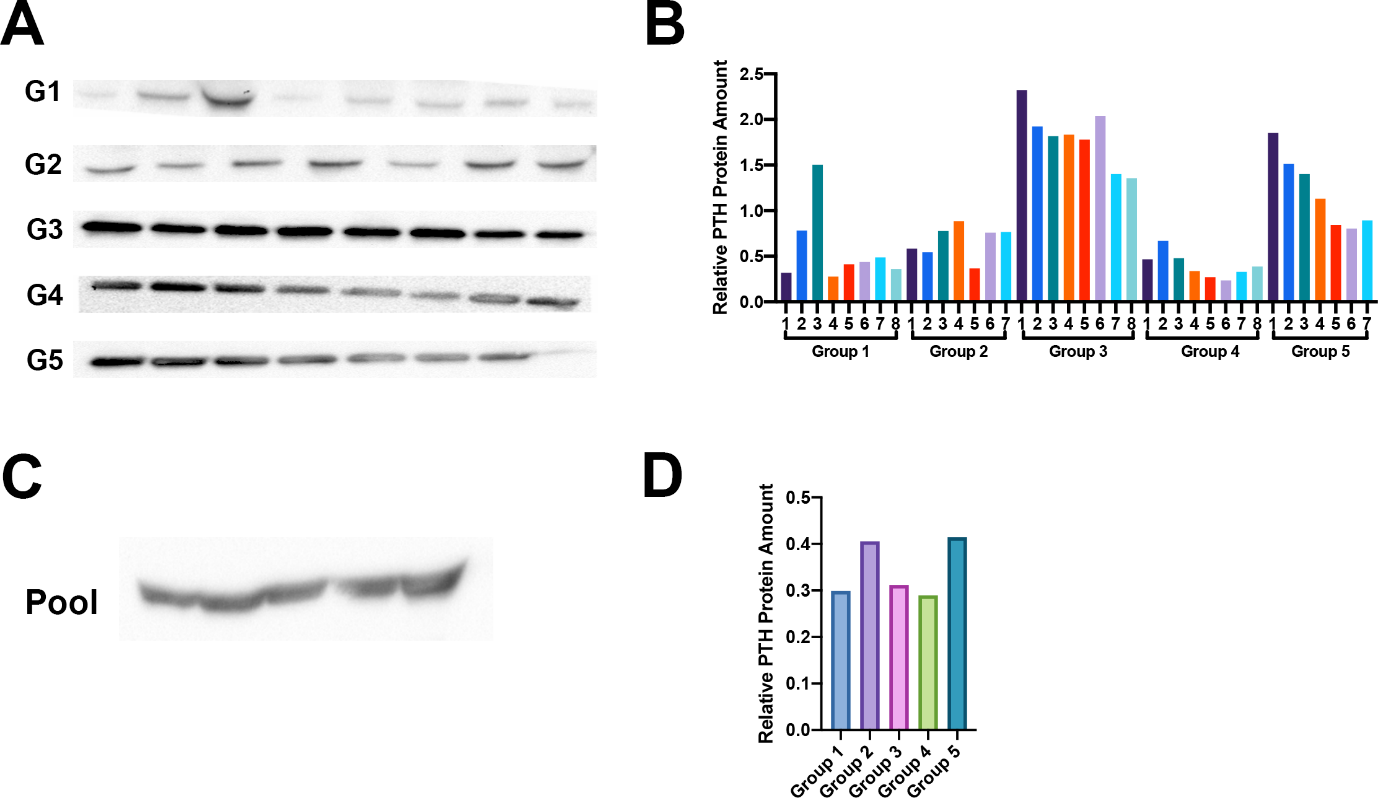


**Supplementary Figure 25.** The quantification of in vivo PTH concentrations in experimental groups. A) Western blot analysis PTH in every rat’s serum. B) The quantity of PTH protein in each rat. C) Western blot analysis of PTH in the pooled serum samples. Data are represented as mean ± SEM. D) The quantity of PTH protein in the pooled serum samples. Data are represented as mean ± SEM.


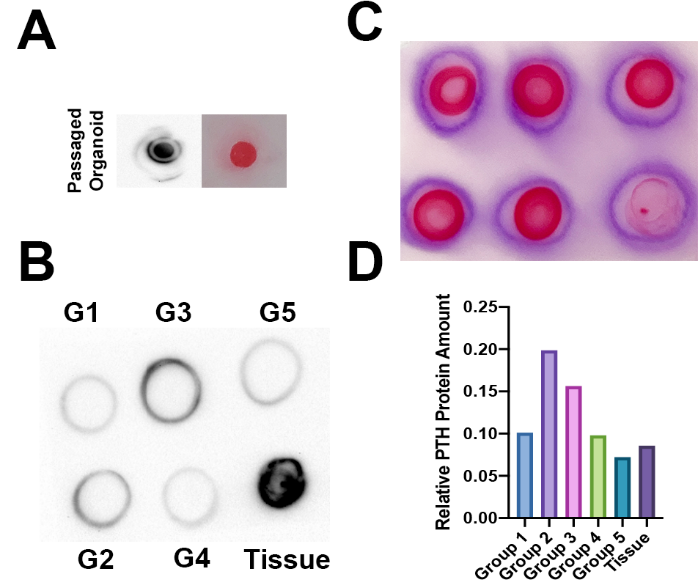


**Supplementary Figure 26.** Dot blot analysis of PTH concentrations in vivo A) PTH concentration of 2 times passaged organoids before transplantation. B) Concentration of PTH in all in vivo groups’ serum and rat parathyroid tissue. C) The utilization of Ponceau S Stain to quantify total protein. D) The normalized relative quantity of PTH with the total protein content.


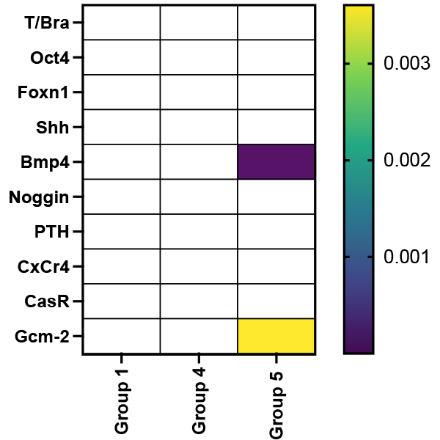


**Supplementary Figure 27.** The gene expression levels of three sets of genes were analyzed in in vivo experimental samples: Set 1 (T/Bra, Oct4, Foxn1); Set 2 (Shh, Bmp4, Noggin); and Set 3 (PTH, CxCr4, CasR, Gcm-2).
