# Supplementary material for: Induced Pluripotent Stem Cell‐Derived Parathyroid Organoids Resemble Parathyroid Morphology and Function
Source: Adv Sci (Weinh). 2024 Sep 27;11(43):2407567. doi: 10.1002/advs.202407567 (PMC11578294; doi:10.1002/advs.202407567)
Supplement: Supplementary file 2 — Supporting Information [file ADVS-11-2407567-s001.docx]

**Supplementary Table 1.** Components of media formulations

| **Media Name** | **Components** | **References** |
| --- | --- | --- |
| Complete Fibroblast Medium | DMEM, 1 g/L glucose  2% FBS  5µg/mL insulin  5 ng/mL bFGF  1% PSA | ^[35}^ |
| N2B27 Medium | Neurobasal medium  DMEM-F12 medium  0.5% N2 supplement  1% B-27 supplement  0.5% of 10% Bovine serum albumin (BSA)  1% L-glutamine  1% PSA | ^[61]^ |
| Osteogenic Differentiation Medium | DMEM, 4.5 g/L glucose  5% FBS  3mM β-glycerophosphate  50 μg/mL ascorbic acid | ^[42]^ |
| Parathyroid Organoid Generation Medium | **(Day 0-Day 10)**  N2B27 Medium  100 ng/mL Activin A  10µM Y-27632  **(Day 10-Day 20)**  N2B27 Medium  100 ng/mL Activin A  10µM Y-27632  50µg/mL Shh | ^[14]^ |

**Supplementary Table 2.** Collected samples for experimental design

| **Experiments** | **Classification of Samples** | **Explanation** | **Experiments** |
| --- | --- | --- | --- |
| *in vitro* organoid generation | D0, D3, D5, D10, D15, D20 | Organoids were collected at day 0-3-5-10-15-20 of differentiation protocol. | IF, WB, qPCR, RNA-Seq, Osteogenic Differentiation, |
| *in vitro* Calcium-induced groups | 2 mM Ca (30min, 1hr)  4 mM Ca  (30min, 1hr) | Day 20 organoids were treated with 2 mM and 4 mM Ca for 30 minutes and 1 hour. | ICP-MS, WB |
| *in vivo* Transplantation | G1, G2, G3, G4, G5 | G1: Control group, G2: Parathyroidectomy group, G3: Parathyroidectomy+PTH injection group, G4: Parathyroidectomy+ORG1 group, and G5: parathyroidectomy+ORG2 group. | IF, IHC, WB, Dot Blot, H&E Staining, ICP-MS, qPCR |

Notes: The term "Org 1" is used for the parathyroid organoids on day 20. Org 2 indicates parathyroid organoids that have undergone two passages.

**Supplementary Table 3.** In vivo experimental groups

| **Groups** | **Number of animals/group** | **Total number of animals used/group** |
| --- | --- | --- |
| **Group1:** Healthy Rat Control Group (Control) | 8 | 8/Control group |
| **Group2:** Parathyroidectomy Group (PD) | 8 | 8/PD group |
| **Group3:** Parathyroidectomy group and parathyroid hormone administration (PD+PTH) | 8 | 8/PD+PTH |
| **Group4:** Parathyroidectomy group and organoid type 1 application (PD+ORG1) | 8 | 8/PD+ORG1 |
| **Group5:** Parathyroidectomy group and organoid type 2 application (PD+ORG2) | 8 | 8/PD+ORG2 |

Notes: The term "Org 1" is used for the parathyroid organoids on day 20. Org 2 indicates parathyroid organoids that have undergone two passages.

**Supplementary Table 4.** Human primer sequences

| **Gene Names** | **Primer Sequences** |
| --- | --- |
| B-Actin | F: 5’ CACCATTGGCAATGAGCGGTTC 3’ |
|  | R: 5’AGGTCTTTGCGGATGTCCACGT 3’ |
| T/Bra | F: 5’ TATGAGCCTCGAATCCACATAGT 3’ |
|  | R: 5’ CCTCGTTCTGATAAGCAGTCAC 3’ |
| Foxa2 | F: 5’ GGGAAATGGGAGGGGTGCAAAAGAGG 3’ |
|  | R: 5’ TTGCGTGAGTGTGGATGGGATTGGTG 3’ |
| Oct4 | F: 5’ TCAGCTGCCAGGAGTGCACG 3’ |
|  | R: 5’ CGCCCTCAGGGAGCAGCCTAC 3’ |
| Shh | F: 5’ CTCGCTGCTGGTATGCTCG 3’ |
|  | R: 5’ ATCGCTCGGAGTTTCTGGAGA 3’ |
| Bmp4 | F: 5’ ATGATTCCTGGTAACCGAATGC 3’ |
|  | R: 5’ CCCCGTCTCAGGTATCAAACT 3’ |
| Noggin | F: 5' CCATGCCGAGCGAGATCAAA 3' |
|  | R: 5' TCGGAAATGATGGGGTACTGG 3' |
| CxCr4 | F: 5' GTGCAGAGTCCAGCAAAGGT 3' |
|  | R: 5' TCAGCCAACTCGTCACAGTC 3' |
| CasR | F: 5' CCACGCATGAGCGGACGCTAA 3' |
|  | R: 5' ATTGGTGGGATGTC TTCGTCTTGG 3' |
| Foxa1 | F: 5' GCAATACTCGCCTTACGGCT 3' |
|  | R: 5' TACACACCTTGGTAGTACGCC 3' |
| Foxn1 | R: 5' CCCTAGCTAGCTAGTAGCTAGT 3' |
|  | F 5’ TATCCCCAACATGCCCATTCG 3’ |
| PTH | F: 5' GAGTAGAATGGCTGCGTAAGA 3' |
|  | R: 5' TTTGTCTGCCTCTCCAAGAC 3' |
| Gcm-2 | F: 5' CTAGAAGAAGCGCCATCAAGAG 3' |
|  | R: 5' AGGGAGGTATGTTGCTGAAATG 3' |
| Sox17 | F: 5' ACAGTATCTGCACTTCGTGTG 3' |
|  | R: 5' GGACACCACCGAGGAAATG 3' |
| ADCY2 | F: 5' TTTATCCTGGTCTGCATCGAGT 3' |
|  | R: 5' TGGCAACAAGGCATATCCATATC 3' |
| CER1 | F: 5' GGATGGCCGCCAGAATCAG 3' |
|  | R: 5' TGGCACTGCGACAAACAGAT 3' |
| ERBB4 | F: 5' GTCCAGCCCAGCGATTCTC 3' |
|  | R: 5' AGAGCCACTAACACGTAGCCT 3' |
| SFRP2 | F: 5' ACGGCATCGAATACCAGAACA 3' |
|  | R: 5' CTCGTCTAGGTCATCGAGGCA 3' |
| AFP | F: 5' AGTGAGGACAAACTATTGGCCT 3' |
|  | R: 5' ACACCAGGGTTTACTGGAGTC 3' |
| ESRG | F: 5' TGGGATGGAGCCATAGAAGT 3' |
|  | R: 5' TGGGTCTTTCAAGAAGTTCCTC 3' |
| FN1 | F: 5' CGGTGGCTGTCAGTCAAAG 3' |
|  | R: 5' AAACCTCGGCTTCCTCCATAA 3' |
| PGK1 | F: 5' TGGACGTTAAAGGGAAGCGG 3' |
|  | R: 5' GCTCATAAGGACTACCGACTTGG 3' |
| Runx1 | F: 5' TTTATCCTGGTCTGCATCGAGT 3' |
|  | R: 5' TGGCAACAAGGCATATCCATATC 3' |
| Col1 | F: 5' TTTATCCTGGTCTGCATCGAGT 3' |
|  | R: 5' TGGCAACAAGGCATATCCATATC 3' |
| Osteonectin | F: 5’ ATGAGGGCCTGGATCTTCTT 3’ |
|  | R: 5’ CTGCTTCTCAGTCAGAAGGT 3’ |

**Supplementary Table 5.** Antibody list

| **Antibody** | **Catalog Number** | **Brand Name** | **Country** |
| --- | --- | --- | --- |
| Brachyruy | sc-374321 | Santa Cruz | USA |
| Foxa2 | ab108422 | Abcam | USA |
| Oct4 | sc-9081 | Santa Cruz | USA |
| Shh | sc-365112 | Santa Cruz | USA |
| BMP-4 | sc-12721 | Santa Cruz | USA |
| Noggin | sc-33759 | Santa Cruz | USA |
| Foxn1 | sc-271256 | Santa Cruz | USA |
| Gcm-2 | sc-390603 | Santa Cruz | USA |
| CasR | ab19347 | Abcam | USA |
| PTH | PA1-28873 | Invitrogen | USA |
| CxCr4 | NB100-56437SS | Novus Biotechnolgy | USA |
| β-actin | 3700S | Cell Signalling Technologies | USA |
| Osteocalcin | ab133612 | Abcam | USA |
| Col1A | ab34710 | Abcam | USA |
| Human Nuclear Antigen | ab191181 | Abcam | USA |
| PECAM1 | 910003 | Biolegend | USA |
| p-ERK | 4370P | Cell Signalling Technologies | USA |
| Erk(1/2) | 9102 | Cell Signalling Technologies | USA |
| Pax6 | sc-514352 | Santa Cruz | USA |
| Sox17 | NBP2-24568SS | Novus Biologicals | USA |
| CD68 | ab283654 | Abcam | USA |
| Alexa Flour 488(Anti-rabbit) | A27034 | Invitrogen | USA |
| Alexa Flour 488(Anti mouse) | A28175 | Invitrogen | USA |
| Goat anti-mouse IgG-HRP | sc-2005 | Santa Cruz | USA |
| Goat anti-rabbit IgG-HRP | sc-2004 | Santa Cruz | USA |
| Donkey anti-goat IgG-HRP | sc-2020 | Santa Cruz | USA |
